# Explainable pulmonary fibrosis detection using edge-strengthened dilated holistic edge detection-based lung segmentation and ResNet-V2 classification

**DOI:** 10.3389/frai.2026.1858838

**Published:** 2026-07-21

**Authors:** K. Mahapackialakshmi, G. Jaffino

**Affiliations:** School of Electronics Engineering, Vellore Institute of Technology, Vellore, Tamil Nadu, India

**Keywords:** chest X-ray, ES-D-HED, explainable artificial intelligence (XAI), lung segmentation, medical image analysis, pulmonary fibrosis, ResNet

## Abstract

**Introduction:**

Pulmonary fibrosis (PF) is a progressive interstitial lung disease that requires accurate and early detection to improve patient survival and treatment planning.

**Methods:**

This study proposes an explainable deep learning framework for pulmonary fibrosis detection from chest X-ray images by integrating an edge-strengthened dilated holistic edge detection (ES-D-HED) segmentation network with a fine-tuned ResNet152V2 classification model. Unlike the conventional HED-based approaches, the proposed ES-D-HED architecture incorporates an additional dilated intermediate-output branch and enhanced multi-scale edge fusion to improve contextual boundary modeling and fibrosis-related structural edge continuity. The framework combines edge-aware lung segmentation, fibrosis classification, and Grad-CAM-based explainability to provide interpretable clinical decision support. The model was evaluated on a curated subset of the publicly available NIH Chest X-ray dataset using patient-level five-fold cross-validation. Since the NIH dataset does not contain fibrosis segmentation masks, representative PF regions were retrospectively annotated by a clinical expert radiologist for quantitative validation.

**Results and Discussion:**

Experimental results demonstrated a classification accuracy of 98.6%, sensitivity of 98.0%, specificity of 99.2%, and F1-score of 98.5%. The proposed segmentation model achieved Dice similarity coefficients of 0.904 for normal lung segmentation and 0.843 for PF region segmentation, indicating strong structural alignment with expert annotations. Grad-CAM visualization also confirmed that the model successfully identified abnormal lung areas associated with fibrosis. The proposed framework shows the potential application of edge-strengthened explainable deep learning in the PF screening system based on chest X-ray imaging. Future studies will involve the classification of multi-class interstitial lung disease, grading the severity of fibrosis, and the multi-center clinical validation for real-world applicability.

## 1 Introduction

A chronic progressive interstitial lung disease that impairs normal lung function and eventually results in mortality, pulmonary fibrosis (PF) damages the lung permanently. A dangerous chronic lung illness called PF causes irreversible lung tissue destruction and scarring in a person's everyday life. Lung function gradually and irreversibly declines over time as fibrotic tissue replaces healthy lung tissue on an increasing basis. Nowadays, PF has limited therapeutic options, and curing the disease remains a significant challenge. The current goals of disease management and treatment are to improve quality of life and slow the rate of lung function decline. A patient's PF progression rate can vary greatly, from little to no change over several years to a rapid decline over a short period. At this stage, PF, a type of interstitial lung disease, remains largely incurable, and understanding its pathophysiology is essential for effective disease management and treatment planning (Cong et al., 2025). Consequently, the main goal of PF treatment is to manage and slow disease progression and deterioration in order to extend the patient's life. Early diagnosis and intervention are critical to prolonging survival in individuals with illnesses such as PF. Consequently, it is critical to get a prompt and precise PF diagnosis (Trusculescu et al., 2020). Several recent studies have emphasized that early identification of PF using artificial intelligence-assisted imaging analysis can improve disease management and clinical decision support (Bernardinello et al., 2025). Imaging, tissue biopsies, and lung function tests are used in the clinical diagnosis of PF. Appropriate precision of the radiology imaging apparatus is necessary for the accurate diagnosis of PF using lung imaging approaches.

Even though high-resolution computed tomography (HRCT) is regarded as the standard for diagnosing PF, its use in clinical practice is less frequent due to increased radiation exposure, increased cost, and low accessibility in low-resource environments. On the other hand, chest X-ray imaging is one of the most ubiquitous and least expensive diagnostic methods worldwide and is commonly adopted as a first-choice screening method. Recent deep learning studies have demonstrated the feasibility of chest X-ray-based PF screening systems for assisting radiologists in resource-limited clinical environments (Chantzi et al., 2025; Kumar et al., 2025). It is possible to develop an artificial intelligence tool to detect fibrosis on chest X-rays, which assists in real-time risk stratification and makes the referral decision for further HRCT. Nevertheless, subtle interstitial variations in X-ray images are difficult to detect due to low contrast and anatomical structures, and therefore, more edge-sensitive segmentation methods are required.

Data preprocessing is a vital step in applying artificial intelligence (AI), significantly impacting the effectiveness and predictive behavior of AI models (Ishwerlal et al., 2024). Raw data undergoes various procedures, including processing, transformation, normalization, integration, reduction, and conversion, to become suitable for training machine learning or deep learning models. In diagnostic imaging, techniques such as computed tomography (CT) and pulmonary X-rays (CXR) are used to visualize the lung's internal architecture, enabling detection of changes and patterns that can inform AI-driven insights. Precise segmentation of lung structures from adjacent tissues and organs is crucial for accurate image interpretation and analysis (Souza et al., 2019). The diagnosis of several lung conditions, such as respiratory nodules, emphysema, lung cancer, and PF, can be aided by pulmonary segmentation. The segmentation of the lungs presents a formidable challenge due to brightness issues, adjacent structures, and bones and other tissues that may obscure the lungs. Computer-aided lung segmentation is a crucial step in the diagnosis and management of pulmonary diseases (Kim and Lee, 2021).

Although there have been recent developments in deep learning-based lung segmentation and classification, existing methods typically rely on fully supervised segmentation with pixel-level fibrosis labels or, in practice, focus on classification and do not explicitly represent the boundary abnormalities. Moreover, few studies have examined the impact of edge-aware modeling on the capture of fibrosis-induced anatomical distortions in chest X-ray images. To overcome such constraints, this research study will suggest an edge-strengthened segmentation model with explainable classification to enhance the structural boundary modeling and clinical interpretability.

Moreover, few studies have examined the impact of edge-aware modeling in capturing fibrosis-induced anatomical distortions in chest X-ray images. The proposed system is intended as a screening support and decision-assistance tool rather than a replacement for HRCT-based diagnostic estimation. The main objective is to assist clinicians in the early identification of potential fibrosis-related structural patterns on chest X-rays, mainly in resource-limited settings where the CT images may not be immediately available. The framework is not designed to provide a standalone clinical diagnosis, nor does it replace the radiologist's interpretation or definitive HRCT-based assessment; instead, it offers supportive structural analysis to enhance preliminary evaluation.

The potential contributions and innovative aspects of this study are as follows:

- A novel Edge-Strengthened Dilated Holistic Edge Detection (ES-D-HED) framework is proposed for lung boundary-aware segmentation in chest X-ray images. The proposed architecture extends the conventional HED network by integrating an additional dilated intermediate-output branch to improve contextual structural edge modeling and the continuity of fibrosis boundaries.
- An enhanced multi-scale feature fusion mechanism is incorporated into the ES-D-HED architecture to strengthen edge representation and improve segmentation consistency in structurally irregular PF regions.
- A fine-tuned ResNet152V2 classification framework is developed by integrating modified dense, batch normalization, dropout, and feature-concatenation layers to improve PF classification performance while reducing overfitting.
- An explainable artificial intelligence framework is integrated using Grad-CAM visualization to provide interpretable localization of fibrosis-related abnormal lung regions and support clinical decision-making.
- The proposed framework integrates segmentation, classification, and explainability within a unified deep learning pipeline for PF screening using chest X-ray images.

The rest of the manuscript is organized as follows: the related studies and literature analysis are presented in Section 2. The proposed framework defines the detection of PF using the ES-D-HED, comprising preprocessing, segmentation, classification, and evaluation metrics, as described in Section 3. The results and discussion are presented under segmentation performance, classification analysis, Grad-CAM explainability, and comparative evaluation, as described in Section 4. Section 5 summarizes the manuscript and provides guidelines for future research.

## 2 Related work

Shamrat et al. (2022) described the classification of many lung diseases, like chest x-ray images that contain PF, X-ray images collected from different resources such as Kaggle, National Institutes of Health (NIH), and GitHub Medical Center, and identification of several lung diseases, such as PF.

Pandurangan et al. (2021) demonstrated that the power-law transformation and adaptive histogram equalization techniques improved the performance of low-light brightness images, enhancing their quality and visibility, particularly in poor illumination. Shape and texture features serve as the foundation for classification in previous methods that classify lung diseases from the entire input image, as found in the existing literature. For example, Hogeweg et al. (2015) propose an automated method for detecting lung illness from the complete set of Chest X-ray pictures. The proposed technique employs a hybrid architecture that integrates multiple subsystems to detect irregularities in shape, texture, and focus within the input image and subsequently classifies the image as either normal or abnormal.

A hybrid-based Bayesian classification method for lung disease categorization was proposed by Shen et al. (2010). This method is proposed to enhance the mean shift segmentation by employing a threshold to segment lung infections and determine the exact contour positions of the infections. Subsequently, the shape and internal boundary of pulmonary infections are taken into account by the Gradient of the Inverse Coefficient of Variation (GICOV) to identify possible lung infections.

The multi-scale rotation-invariant along with the CNN algorithm and its core were proposed by Wang et al. (2018). To simulate the visual cortex of a human, the scientists employed a filter, called Gabon, that can examine specific frequencies and directions within a limited area. In categorizing PF patterns like GO & MN, the suggested approach achieved up to 90% accuracy. With minimal fibrosis localization data, Li et al. (2018) classified and localized the region of anomaly using the ResNetv2 structure. A modified version of the Deep CNN framework, MobileNet V2, was developed by Souid et al. (2021) to identify and classify lung diseases, including PF, from anterior thoracic X-ray scans. According to the report, the authors classified PF-positive X-ray images with 96.6% accuracy and 47.2% sensitivity. Li et al. (2022) predicted lung fibrosis with 93% accuracy using the chest X-ray 14 dataset and Inception-ResNet v2 architecture.

Xie and Tu (2015) introduced the revolutionary end-to-end edge deep model HED, which used side and fusion branches to calculate losses and generate the concluding edge maps. Anand et al. (2023) proposed two optimizers, i.e., Adam & Adadelta, to analyze the model for skin disease classification. The U-Net model accurately localizes the Region of Intersection (ROI), whereas the CNN model achieves multi-class classification on segmented images. These dual optimizers enhance model performance, achieving an accuracy of 97.96% and outperforming the Adadelta optimizer.

Edge detection for forensic dentistry was proposed by Jaffino and Prabin Jose (2021), who proposed that the contourlet transform-based edge detection technique can effectively detect edges and that similarity-based matching is used in forensic dentistry. The new mathematical modeling-based contour shape extraction was proposed by Jaffino et al. (2020), and an Online Region-based Active Contour Model (ORACM) for form extraction.

Baltruschat et al. (2019) conducted a comparative analysis of various deep learning (DL) architectures for multi-label X-ray classification, revealing that ResNet-38, a model optimized for X-ray images, demonstrated superior performance in fibrosis classification, achieving the best overall results. According to Spira et al. (2004), smoking remains a significant risk factor due to its potential to cause irreversible lung injury, leading to long-term damage and chronic health issues. Additionally, the prognosis of idiopathic pulmonary fibrosis (IPF) patients who have ever smoked is worse than that of non-smokers. Numerous epidemiological investigations have indicated that environmental exposures contribute to the pathophysiology of IPF. All the findings have strongly linked metal dust and cigarette smoking to the incidence of IPF, particularly for the familial variant of PF, despite the lack of evidence of a dose-response association.

Demir et al. (2020) mainly focused on automatic lung disease analysis using deep learning techniques for lung sound classification. Some of the CNN models for feature extraction and classification include AlexNet, ResNet-50, and VGG-16. The proposed methods aim to improve classification accuracy for the automatic detection of lung disease. Islam and Zhang (2018) emphasized the importance of automated lung segmentation in computer-aided X-ray image analysis, highlighting its role in facilitating accurate recognition and diagnosis. But it is difficult due to edges, irregular shapes, and the appearance of the lung apex. The suggested model learns to disregard regions and highlight the valuable ones for lung segmentation. It achieves a strong Dice score of 98.6% using two publicly available datasets.

Ioffe and Szegedy (2015) presented a normalizing layer to overcome batch normalization. Batch normalization permits a higher learning rate, disregards the need for dropout, and enables cautious initialization. Batch normalization produces advanced results in ImageNet categorization. Candemir et al. (2014) presented a robust lung segmentation technique for digital chest X-rays. It is used to automatically detect lung regions, particularly in digital chest X-ray images. This technique uses image retrieval, nonrigid registration, and graph-cut optimization to identify lung boundaries with more accuracy, achieving a 95.4% improvement in performance. Szegedy et al. (2016) emphasized that, for mobile visualization and high-data scenarios, reducing parameter counts and enhancing computational efficiency are crucial. This study achieves efficient scaling of systems, resulting in state-of-the-art performance on the ILSVRC 2012 classification challenge, with significantly reduced computational cost and fewer parameters.

Kaur et al. (2017) described a deep learning-based CNN that is used to achieve outperform for many existing systems in the lung field segmentation method and all of the CNN model layers are achieved with enhanced accuracy, overlap, sensitivity, and specificity. Rashid et al. (2018) described a deep neural network to achieve improved performance of machine vision comparable to humans. The proposed methodology was tested with the dataset images and gave high accuracy for medical imaging segmentation tasks. Reza et al. (2020) proposed a new model for lung segmentation, i.e., TransResUNet, which enhanced the advances of the U-Net model. This achieved superior performance and outperformed the starting point U-Net model with an accuracy of 98.5% and a Dice coefficient of 97.6%. Hence, this model a highly effective and efficient solution for lung segmentation. Salisbury et al. (2016) explained that in certain patients with interstitial lung disease (ILD) and HRCT fibrosis but no honeycombing, IPF can be detected without a surgical lung biopsy. This can be particularly useful when older people (above 60) are combined with radiologic disease severity.

To improve the quality of life, two antifibrotic drugs, pirfenidone and nintedanib, are used. Pirfenidone reduces progression and improves survival, assisting in IPF management (Glassberg, 2019). Malialis et al. (2022) proposed the Augmented Queues method, which improves learning quality and speed in data stream classification. Nishikiori et al. (2023) established a deep CNN to detect CT-confirmed CF-ILD from chest x-rays. The model achieved high diagnostic performance. and was statistically superior to pulmonologists and radiologists (*p* < 0.01). It remained robust across validation datasets and could detect fibrosis involving as little as 0–10% of the lung area.

Maruthai et al. (2025) demonstrated that the MaxTU-CBE mitigates CNN localization bias in CXR classification via transformer-enhanced semantic segmentation. This model integrates hierarchical multi-axis transformers into the encoder and decoder pathways of a U-Net architecture, establishing global feature dependencies via a contextual fusion engine (CFE) utilizing self-attention.

Table 1 compares existing lung segmentation and classification approaches. In real-time analysis, obtaining high-quality fibrosis-specific masks can be rare and challenging. Additionally, the structural boundary abnormalities induced by fibrosis are not explicitly modeled in many studies, which predominantly emphasize classification tasks. Holistically-nested edge detection (HED) is also a generic edge detection technique with an edge-aware design, but its application directly focuses on lung fibrosis segmentation without architectural adaptations to address atypical boundary discontinuities caused by interstitial pathology, which may limit its effectiveness in identifying fibrosis. Hence, it is still necessary to have an edge-strengthened segmentation model with better sensitivity for segmenting the edge boundaries of the fibrosis region, and that incorporates structural modeling with explainable classification.

**Table 1:** Comparison of existing lung segmentation and classification approaches.

References	Dataset used	Model	Annotation requirement	Explain-ability	Limitation	Future scope
Souid et al. (2021)	Chest X-ray	MobileNetV2	No segmentation	No	No structural modeling	Explainable fibrosis localization
Li et al. (2022)	NIH Chest X-ray	Inception-ResNetV2	No segmentation	No	No boundary modeling	Edge-aware fibrosis detection
Reza et al. (2020)	Chest X-ray	TransResUNet	Full supervision	No	High complexity	Lightweight segmentation models
Rana et al. (2025)	Chest X-ray	IMSTrans	Full supervision with augmentation for imbalance	No	Generalization to rare diseases	Multi-center validation
Fu et al. (2025)	Chest X-ray	LungMaxViT	Full supervision (image-level multi-label annotations)	Yes	Computational cost of transformers	Lightweight explainable transformer
Ahmad et al. (2025)	Chest X-ray	ViT and swin transformer	Full supervision (binary/multi-class image labels)	No	Less focus on the subtlety of fibrosis	Structural abnormality modeling
Toshniwal et al. (2025)	Chest X-ray	Clinical case analysis	No segmentation	No	Case series (*n* = 2); no AI/ML; descriptive only	Develop AI-based pneumothorax detection in ILD; add Grad-CAM for CXR interpretation
Gao et al. (2026)	Chest X-ray	Multi-modal transformers	Full supervision	No	Primary dataset is HRCT-based; limited ILD patterns; requires paired CXR-CT	CXR-first screening workflow; real-time report generation; integrate with staging
Proposed ES-D-HED	NIH Chest X-ray	ES-D-HED + ResNet152V2	Representative expert annotation	Yes	Limited to binary classification	Multi-class ILD grading

Current detection methods for PF primarily aim to classify patients with classification accuracy, with minimal attention to fibrosis-specific structural boundary modeling and explainability. Most of the existing approaches also rely on fully supervised annotations and complex architectures that require excessive computational effort. The proposed ES-D-HED framework is motivated by the above limitations and combines edge-strengthened segmentation, contextual boundary modeling, and explainable classification into a single architecture.

## 3 Proposed methodology

A deep learning algorithm is employed to identify PF in chest radiographic images, enabling accurate detection and classification of the disease. The X-ray image is a two-dimensional image created by X-rays that pass through human tissues and organs, which are then projected onto a particular negative image that allows the lesions to be seen in various positions. One of the most popular and economical medical imaging exams in medical practice is the chest X-ray.

### 3.1 Overview of the proposed framework

This study focuses on improving the detection of PF in chest X-ray images by performing a series of enhancement and edge-aware segmentation operations. It proposes a framework that performs some pre-processing operations, such as filtering, log normalization, and gamma correction, to enhance the structural visibility and consistency in contrast prior to segmentation. The improved images are then analyzed with the proposed ES-D-HED architectural approach for fibrosis-sensitive lung boundary modeling and classification.

**Figure 1:**
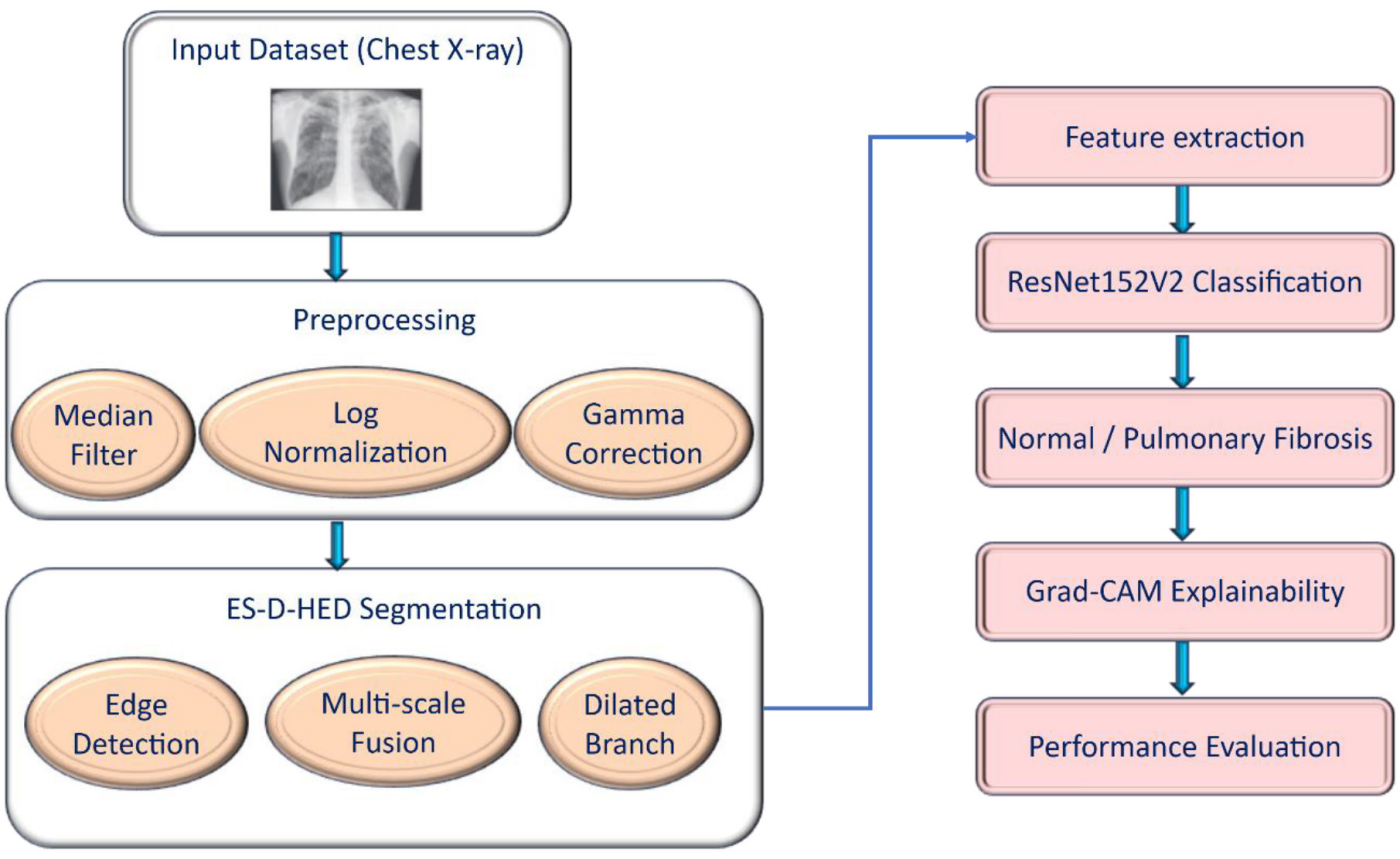
Workflow of the proposed ES-D-HED and ResNet152V2-based binary classification framework.

As shown in Figure 1, the process begins with data collection in which lung images are gathered from publicly available sources. Next, preprocessing techniques are applied to clean, enhance, and transform the images into a format suitable for computer-aided analysis. The improved images are then analyzed using the proposed ES-D-HED segmentation framework to extract fibrosis-sensitive structural lung boundaries. The segmented outputs are then classified using a fine-tuned ResNet152V2 architecture, and explainable region localization is achieved by Grad-CAM visualization of fibrosis-related regions. Lastly, the framework is tested for its performance with segmentation and classification metrics.

### 3.2 Dataset description

The dataset used in this study corresponds to the NIH Chest X-ray dataset, which is a collection of 112,120 frontally viewed chest X-ray images of 30,805 different patients. The data was obtained from the publicly available repository hosted on Kaggle. The data is publicly accessible and was conveniently retrieved from its Kaggle repository. In this research, there was the creation of a curated subset of cases of normal and PF only. Following the filtering and class balancing, 3,372 images were used in the final dataset, comprising 1,686 normal and 1,686 PF images. To ensure a strict experimental design and avoid data leakage, data splitting was done at the patient level. The images of a particular patient were divided only into the training, validation, and testing sets. The data was separated into training (70%), validation (15%), and testing (15%) during the maintenance of the class-balanced dataset. Table 2 the dataset distribution. All dataset partitioning and cross-validation methods were executed at the patient level to prevent data leakage. Images associated with the same patient were not allocated across several subsets. The resolution of the X-images was adjusted to 224 × 224 pixels.

**Table 2:** Dataset distribution.

Dataset split	Normal	PF	Total
Training (70%)	1,180	1,180	2,360
Validation (15%)	253	253	506
Testing (15%)	253	253	506
Total	1,686	1,686	**3,372**

Bold values indicate the total counts.

As the NIH dataset does not include fibrosis-related segmentation masks, a representative sample of the PF images was retrospectively annotated by a board-certified radiologist with expertise in imaging. Areas of fibrosis were outlined based on radiographic findings, such as reticular appearances and interstitial structural deformity. Such annotations were applied only to the quantitative assessment of the structural alignment of predicted segmentation maps and clinical expert-defined regions.

### 3.3 Image preprocessing

All chest X-ray images were preprocessed before segmentation and classification to enhance the visibility of the structures and the consistency of their contrast. The median filter was used to minimize artifacts (Ishwerlal et al., 2024), and log normalization was applied to normalize the intensity (Pandurangan et al., 2021). The fibrosis-sensitive structural regions were then enhanced using gamma correction (γ = 0.6). Finally, all the images were resized to 224 × 224 pixels and normalized prior to segmentation using ES-D-HED. The preprocessing function is calculated using Equation 1,


$$\begin{array}{l} P\left(I_{\left(x\text{\_}ray\right)}\right)=\;I_{\left(x\text{\_}ray\right)Enhance}\left(x,y\right) \end{array}$$ (1)

Where, *P*(*I*_(*x*_*ray*)_) is the pre-processed image. To enhance the input image quality and diagnostic comprehensibility of X-ray images for PF detection, a sequence of median filtering, log normalization, and gamma correction is applied in the preprocessing pipeline. The preprocessing steps include median filtering with a 3 × 3 kernel to reduce the noise. Log normalization and gamma correction were used to enhance the image contrast. These pre-processed outputs *P*(*I*_(*x*_*ray*)_) are suitable for extracting the features and classifying lung images. The mathematical expressions of median filtering and the enhanced function of gamma correction are given by Equations 2, 3,


$$\begin{array}{l} I_{\left(x\text{\_}ray\right)D}\left(x,y\right)=MedianF\left(I_{\left(x\text{\_}ray\right)N}\left(x,y\right)\right) \end{array}$$ (2)

$$\begin{array}{l} I_{\left(x\text{\_}ray\right)Enhance}\left(x,y\right)=\;{(\frac{log(1+I_{\left(x\text{\_}ray\right)D}\left(x,y\right)}{log(1+I_{(x\text{\_}ray)\max})})}^{\gamma } \end{array}$$ (3)

Where *I*_(*x*_*ray*)*D*_(*x, y*) is a denoised image; *I*_(*x*_*ray*)max_ is the maximum pixel value; *I*_(*x*_*ray*)*Enhance*_(*x, y*) is the enhanced pixel value; γ represents the Gamma transformation. This gamma transformation enhances contrast between the different lung tissues and makes abnormalities more visible. For this purpose, the gamma value γ = 0.6 is used to enhance the images. The gamma transformation is useful to improve the visibility of lung abnormalities by regulating image contrast. In this method, the enhancement is done using log normalization and gamma correction. Initially, the log normalization compresses the dynamic range of pixel intensities, and then gamma correction is used to boost specific intensity regions.

### 3.4 ES-D-HED-based segmentation

Image segmentation, feature extraction, and classification are required to identify lung diseases. Segmentation is used to highlight lung images by dividing them into different regions of interest (ROI). To detect lung disorders, pertinent features such as the size, shape, and structure of lung nodules are extracted from images. Classification is used to categorize the image as either normal or abnormal based on the extracted features, facilitating the detection of lung diseases. Image examinations use computer-aided analysis to scrutinize the image and identify potential lung diseases, enabling accurate diagnosis and timely intervention.

Image analysis is used to identify lung disease through segmentation, feature extraction, and classification. Here, segmentation is used to highlight the lung in the image by partitioning it into distinct regions of interest. Feature extraction continues with the detection of lung disorders, where pertinent characteristics such as the size, shape, and structure of lung nodules are identified. Then, using these extracted features, images are classified as either abnormal or normal, enabling the identification of lung conditions.

A deep learning model called edge strengthened dilated holistic edge detection (ES-D-HED) uses both deep supervised nets and fully convolutional neural networks (CNN) to detect edges in unlabeled images. ES-D-HED is a deep-learning architecture for edge detection and image segmentation. Deeply supervised nets serve as the model for nested multi-scale feature learning using deep-layer supervision to “guide” early classification outcomes. ES-D-HED is a potent edge detection method that produces precise, in-depth edge predictions using deep supervision and fully convolutional network (FCN) capabilities.

Edge strengthened dilated holistic edge detection (ES-D-HED) is a powerful technique for image analysis that, rather than focusing only on local patches, identifies edges in an image. As a result, it can record edges more robustly and accurately. When analyzing X-ray images to detect PF, ES-D-HED can be very helpful in the following situations: sensitive edges and patterns in the lung tissue, early detection of fibrosis symptoms such as reticulation or ground-glass opacities, and segmentation of lung areas and anomaly detection. Preprocessed images are analyzed with a multistage holistic edge detection Equation 4 given below,


$$\begin{array}{l} ESDHED\left(\;P\left(I_{\left(x\text{\_}ray\right)}\right)\;\right)=\nabla \left(\;P\left(I_{\left(x\text{\_}ray\right)}\right)\;\right)⊗K \end{array}$$ (4)

**Figure 2:**
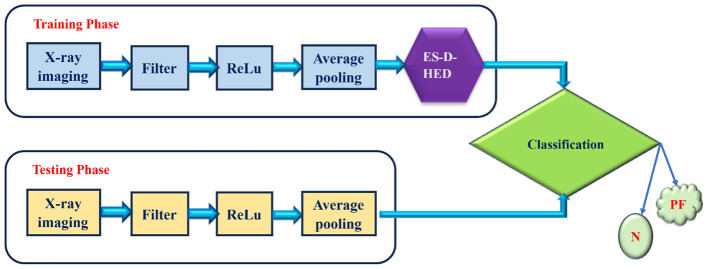
General architecture structure for image classification. N, normal; PF, pulmonary fibrosis.

Where *P*(*I*_(*x*_*ray*)_) is the input image; ∇ is the gradient operator; ⊗ *is denoted as convolution operator*; *and* *K* is represented as the kernel. A five-fold cross-validation setup with preprocessed images ensures robustness and minimizes overfitting, improving the model's performance. The image sizes are standardized to ensure an even input size before proceeding to the next detection stage. As shown in Figure 2, the ES-D-HED framework consists of training and testing phases. For this purpose, the input X-ray images, a filter, ReLU, and a pooling function are used to improve the model performance and disease classification in normal and abnormal conditions. The ES-D-HED framework requires several key preprocessing steps to ensure optimal feature representation and edge detection performance. The main key preprocessing steps are filtering, ReLU, and average pooling. Filters are used to reduce noise and level the image, which improves edge detection by removing irrelevant high-frequency components. The ReLU is used to suppress negative values and conserve activated features. Average pooling reduces the spatial resolution while retaining essential information. So ES-D-HED ensures the performance of segmentation by providing clear, more structured edges from the input X-ray images for accurate edge detection. From these results, the testing data can identify the normal and abnormal lungs, and then it can easily classify the X-ray images as normal or PF. This ES-D-HED framework is further enhanced and illustrated using an architecture diagram.

The implementation of the ES-D-HED model includes distinct training and testing phases to optimize and evaluate its performance, respectively. The structure of ES-D-HED consists of multiple convolutional layers, a holistic edge refinement module, and a multi-scale feature extraction mechanism. Formulate the ES-D-HED approach for the edge detection training phase of the input data set denoted by Equation 5,


$$\begin{array}{l} S=\left\{\left(x_{m}\;,\;y_{m}\right),\;m=1,2,3,\ldots \ldots \ldots ..M\;\right\} \end{array}$$ (5)

$$\begin{array}{l} x_{m}=\left\{{x_{j}}^{\left(m\right)}\;,\;j=1,2\ldots \ldots \ldots \ldots \ldots \ldots \ldots \ldots \ldots |x_{m}|\right\} \end{array}$$ (6)

$$\begin{array}{l} y_{m}=\left\{{y_{j}}^{\left(m\right)}\;,\;j=1,2\ldots \ldots \ldots \ldots \ldots \ldots \ldots \ldots \ldots |y_{m}|\right\} \end{array}$$ (7)

$$\begin{array}{l} y_{j}^{\left(m\right)}\;\text{£ }\left\{0,1\right\} \end{array}$$ (8)

*x*_*m*_ and *y*_*m*_ denotes the collected input images and the binary edge map for the image *x*_*m*_. Equation 6 describes the total number of inputs and ground truth of the corresponding input with different sets of data. Equation 7 represents the matching true labels binary edge map for the input image *x*_*m*_. The network learns features from given input images to create edge maps with labels for segmentation as defined in Equation 8. Segmentation output is associated with the classifier. The objective function described in (Xie and Tu, 2015) and the segmentation Equation 9 given below,


$$\begin{array}{l} R_{seg}\left(R,r\right)=\overset{R}{\underset{r=0}{\sum }}\alpha_{r}\;{r_{seg}}^{\left(r\right)}{(R,r}^{(r)}) \end{array}$$ (9)

*R*_seg_ represents the loss function of the segmentation output. *R* denotes the layer of the network in standard form. r denotes the layer weight, i.e., *r* = (*r*^(1)^,………*r*^(R)^). This process is computed in all the pixels in the images of the training. The collected image data is normalized by dividing pixel values by 255, and the images are read as NumPy arrays to facilitate matrix-based processing. ES-D-HED is a faster edge detection method that enhances detection in less time. This is the major advantage of ES-D-HED. The loss function can easily separate the classes as normal and abnormal with the help of a classifier to give better results. To compensate for the average accuracy being below 0.5%, the flattened layer output is combined with the segmentation outputs from ES-D-HED, thereby enhancing overall accuracy.

During testing time, the collected input X-ray images are used to obtain the edge map identification from both the weighted layer ( Ẑ_*fuse*_) and lateral layer (Ẑ_*seg*_). To formulate the MS-HED approach for edge detection, while the testing phase of the input data set is denoted by the Equation 10,


$$\begin{array}{r} \left({\;Ẑ}_{fuse},\;{Ẑ}_{seg}^{\left(1\right)}\ldots \ldots \ldots \ldots \ldots \ldots \ldots .{Ẑ}_{seg}^{\left(R\right)}\right)=\\ {CNN(x(R,r,h)}^{2^{*}}) \end{array}$$ (10)

Where CNN(·) term represents the edge mapping of an x-ray lung image. Multiple layers are added and extend the weighted layer & lateral layer to get improved performance of segmented image noted as 2^*^ in the Equation 10. The final results can be taken as the average value of the training output of all epochs, obtained by generating an edge map to produce single and multiple enhanced outputs using Equation 11.


$$\begin{array}{l} {Ẑ}_{ES-D-HED}=AVG\left({\;Ẑ}_{fuse},\;{Ẑ}_{seg}^{\left(1\right)}\ldots \ldots \ldots \ldots \ldots \ldots \ldots .{Ẑ}_{seg}^{\left(R\right)}\right) \end{array}$$ (11)

To enhance boundary accuracy in lung area segmentation, the method expanded the holistically-nested edge detection framework into a multi-task segmentation network. MS-HED was initially developed for edge detection utilizing intermediate outputs at various scales. This model incorporates an additional segmentation branch, the ES-D-HED edge branch, in our methodology. This study utilizes ES-D-HED-generated edges for precise border supervision, while the segmentation branch ensures regional uniformity. These components collaborate to create clean lung masks with clearly defined boundaries, facilitating accurate identification of disease-affected areas.

**Figure 3:**
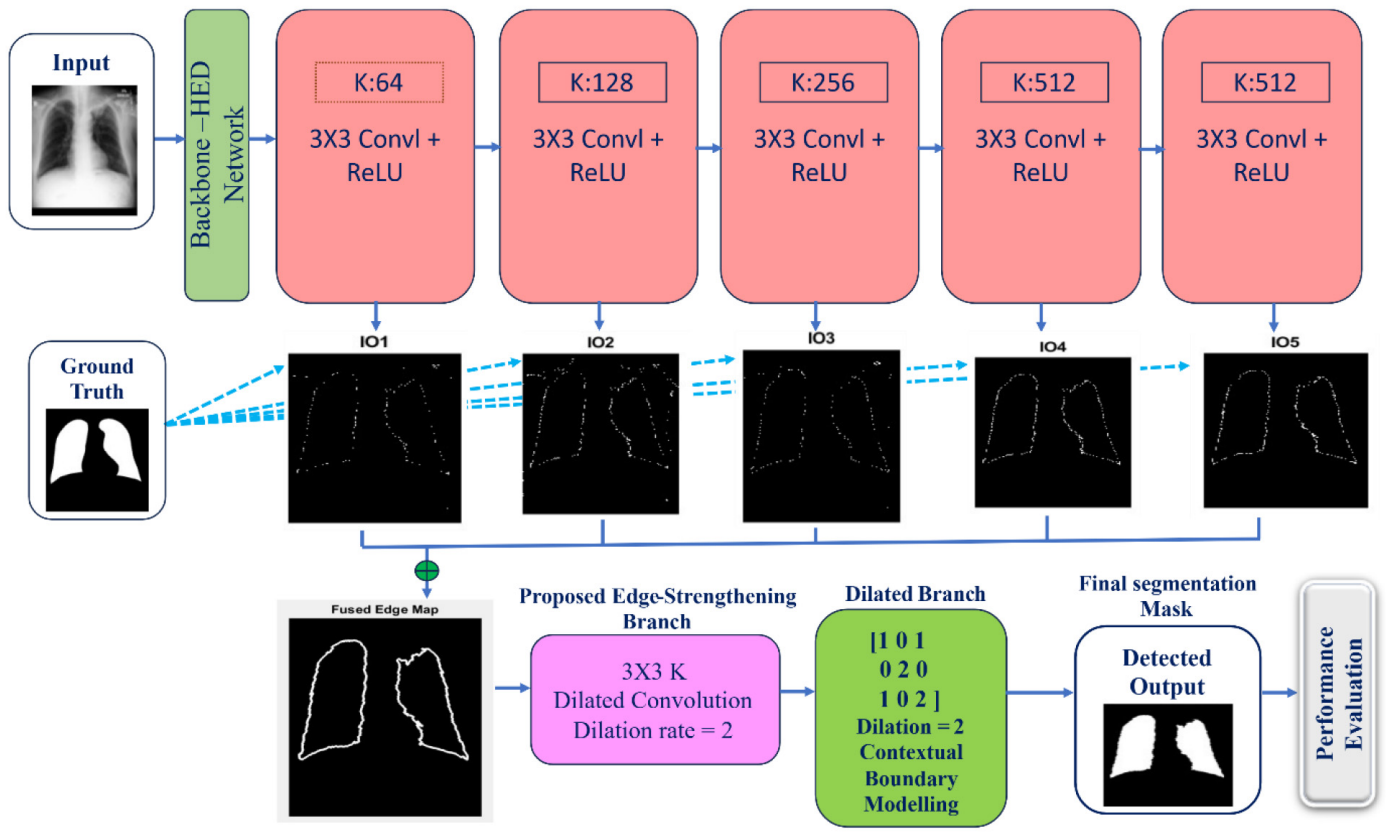
Proposed ES-D-HED architecture of multi-scale intermediate outputs, fused edge representation, and the dilated edge-strengthening branch for contextual lung boundary modeling and pulmonary segmentation.

The multi-task HED architecture comprises convolutional blocks, an edge detection branch, and a segmentation branch. This proposed model initiates with a framework consisting of convolutional blocks that extract multi-scale information from the input image. The features are processed through two concurrent branches: an edge detection branch employing holistically-nested edge detection, which produces intermediate outputs that are fused to create the final edge map, and a segmentation branch that uses a decoder to construct the final lung mask. This model employs a joint loss function that integrates edge and segmentation loss to enhance both the structural boundary precision and region-level segmentation efficiency. ES-D-HED uses a multistage, holistically nested architecture that combines features at various scales and purposes to detect the edges shown in Figure 3. This is achieved through the use of convolutional layers, fusion layers, lateral outputs, and the resultant classified segmentation output. The extra added convolutional layers are utilized to get the two different segmentation outputs for the signal input images. This Dice score similarity value is used to compare with the label image by the fusion layers. Then, from the lateral output segmentation image, which one of the DSC is matched with the label images that are detected as output segmentation? That is one part of architecture where modifications are made using ES-D-HED.

The proposed dilated convolution block is incorporated after Stage 5 of the HED network. The block utilizes a 3 × 3 convolutional kernel with a dilation rate of 2 and padding of 2, so enlarging the receptive field without augmenting the kernel size. The supplementary dilation layer produces a new intermediate output (IO6) that encompasses more contextual information about lung boundaries and fibrosis edges, thereby enhancing the ultimate segmentation performance.

The proposed system first enhances edge detection with CNN-based preprocessing and then performs classification on the segmented output, incorporating key architectural modifications to improve accuracy. The structure is derived from the ES-D-HED architecture, where each layer contributes to improved edge detection and overall system performance. Multi-stage holistic edge detection detects better, coarser edges of the lung region by preprocessing the images at multiple scales. Next, lateral segmentation represents the concatenation, and the transpose layers are utilized. It combines multi-level feature maps and aligns them to the original resolution using transpose. This helps reconstruct the high-resolution segmentation masks from features. After that, the segmentation output produces a probabilistic lung mask and highlights the lung regions. Finally, ES-D-HED detects the input images as normal and PF from the structure of the lung regions. The ES-D-HED approach is used to improve structural edge representation in chest X-ray images, enabling boundary-aware segmentation of lung areas and modeling structural abnormalities associated with fibrosis. This segmentation module facilitates downstream classification rather than serving as an independent pathological segmentation method. Edge disruption analysis serves as a structural indicator, and it is enhanced by deep feature categorization, rather than being an independent diagnostic criterion.

The proposed ES-D-HED enhances the original holistically-nested edge detection (HED) framework by integrating a dilated intermediate-output branch that expands the receptive field without augmenting parameter complexity. In contrast to the conventional HED architecture, which uses fixed convolutional receptive fields for edge prediction, the suggested design incorporates dilated convolution in the deeper intermediate-output layer to get more extensive contextual structural information. In contrast to multi-scale HED (MS-HED), which primarily consolidates multi-resolution edge responses, ES-D-HED prioritizes edge enhancement through dilatation to enhance boundary continuity in structurally irregular lung areas. This alteration is especially pertinent for the diagnosis of PF because interstitial anomalies present as diffuse and spatially extensive structural deformities instead of clear, discrete boundaries. Table 3 compares HED variants.

**Table 3:** Conceptual comparison between HED variants.

Model	Multi-scale supervision	Dilated convolution	Edge strengthening strategy	Designed for PF structural modeling
HED	Yes	No	Standard edge detection	No
MS-HED	Yes	No	Multi-resolution aggregation	No
ES-D-HED (proposed)	Yes	Yes	Dilated receptive field for contextual edge modeling	Yes

Pulmonary fibrosis is identified by widespread reticular patterns and interstitial structural deformation that span extensive areas. Dilated convolution expands the receptive area, allowing the model to detect contextual boundary discontinuities linked to fibrotic tissue. This improves the identification of structurally anomalous lung regions relative to conventional convolutional edge detection.

### 3.5 ResNet152V2-based classification

The deep learning method describes image classification for lung images in top PF and normal. Transfer learning is applied using pre-trained models to fine-tune four architectures, DenseNet-121 (Arulananth et al., 2024), DenseNet-201 (Wang and Zhang, 2020), VGG16 (Sitaula and Hossain, 2021), ResNet-50 (Elpeltagy and Sallam, 2021), and ResNet152v2 for feature extraction and disease detection from a chest X-ray image dataset. With in-depth learning, these models achieve state-of-the-art performance on different image classification tasks. Thus, for lung disease classification, all models are trained and validated to compare results and identify the enhanced performance of the proposed model, i.e., ResNet152V2, which achieves higher accuracy among these pre-trained models after fine-tuning. During the evaluation period, the model achieved higher accuracy but also overfit. To address overfitting, K-Fold cross-validation is used to rectify the issues.

**Figure 4:**
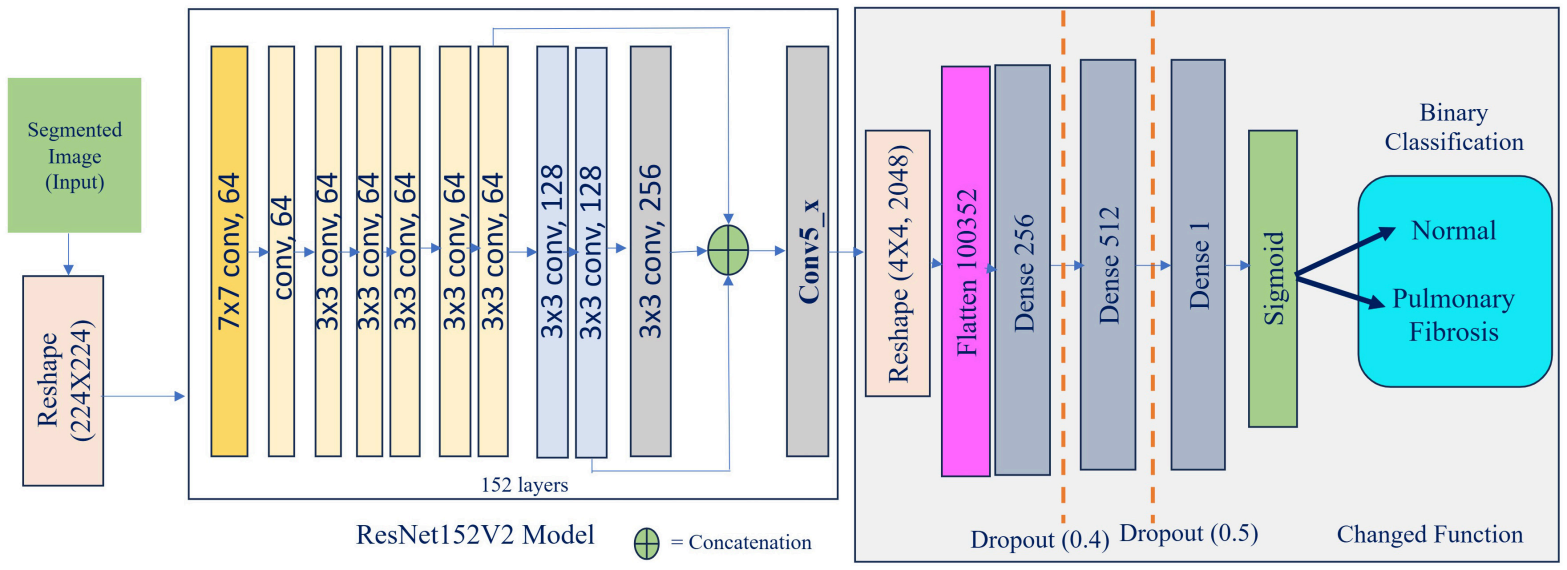
Proposed model for classifying disease.

In this study, transfer learning is applied to five variants of pre-trained models: DenseNet, VGG, and ResNet. These variants were initially trained on the ImageNet benchmark dataset. The models, as mentioned above, undergo explicit fine-tuning on X-ray images from the PF detection dataset. The architecture of the fine-tuned ResNet152V2 is depicted in Figure 4. The base model is initialized with ImageNet-trained weights as a starting point for fine-tuning the pre-trained ResNet model. Each block's convolutional base remains unaltered, but a pooling layer is added atop the ResNet framework to reduce dimensionality. Without sacrificing accuracy, the pooling layer simplifies the network by lowering the number of parameters.

The Conv4_x and Conv5_x are deeper layers that capture high-level features of lung diseases and complex textures. To enable skip connections between Conv4_x and Conv5_x, downsample the Conv4_x output to match the spatial dimensions of Conv5_x, and apply a 1 × 1 convolution on Conv4_x to match the 2048-channel depth of Conv5_x. The transpose of the convolution layer is employed to restore spatial resolution, and the intermediate convolution-extracted features are concatenated and used to classify whether the input is normal or fibrosis, using a sigmoid function for binary classification. This approach helps improve overall classification performance.

After that, a flattening layer is added. The data is then activated using the rectified linear unit (ReLU) activation function after passing through a dense layer with 128 hidden units. The dense layer is followed by a dropout layer with a 50% dropout rate to reduce overfitting. Finally, detections corresponding to the given labels are produced using a dense layer with a single unit. A fresh set of weights and biases is applied to each feature map, resulting in a linear transformation that produces probabilities. The end classifier used for the task is a sigmoid activation function. The PF detection dataset is used to retrain the complete architecture.

The convolutional layers use several scales to extract the characteristics from the input image. Batch normalization, convolutional filters, and ReLU activation functions are used for each convolutional layer. The edge detection findings from various scales are combined in the fusion layers. A sigmoid activation function and a convolutional layer comprise each fusion layer. The edges are identified with several scales of the lateral outputs. Each intermediate output consists of a sigmoid activation function and a convolutional layer. These functions are the key components of the architecture. The convolution layer is responsible for processing and feature extraction, and sigmoid activation is used for output normalization.

Training the ES-D-HED network uses a combination of loss functions. Loss functions are used for segmentation and edge detection. In this study, the soft intersection over union (IoU) loss function is used for segmentation. By predicting precise margins, the model is encouraged to optimize segmentation, resulting in more accurate and refined segmentation. The segmentation edges are defined using the Equation 12,


$$\begin{array}{l} E\left(x,y\right)\;=\;\sum \left(\nabla I\left(x+i,y+j\right)*w\left(i,j\right)\right) \end{array}$$ (12)

Where *E*(*x*,*y*) represents the edge map, with a range of (−1,1) because −1 represents no fibrosis and 1 represents fibrosis; *w*(*i*,*j*) represents weights for the neighborhood, and both variables are used to improve the performance of the loss function; *i* is denoted as the previous layer; *j* is denoted as the current layer. For the edge map, need to strengthen the edges using the gradient magnitude equations for single-stage and multi-stage edge detection Equation 13 given below,


$$\begin{array}{l} \nabla I\left(x,y\right)\;=\;\surd \left(\left(\partial I/\partial x\right)+\left(\partial I/\partial y\right)\right) \end{array}$$ (13)

Where *I*(*x*,*y*) denotes the image intensity and *x*,*y* represent the directions of the image; ∂*I*/∂*x* & ∂*I*/∂*y* represent the single- and multi-stage partial derivatives. Based on Equations 12, 13, images can detect enhanced edge strength to identify the perfect segmentation map and extract pertinent features, such as abnormalities or lung regions.

Classification is used to divide the X-ray images into two classes. That is normal; normal X-ray images show no signs of PF, whereas abnormal X-ray images indicate the presence of PF. This function is described in the class label, which is written in Equation 14,


$$\begin{array}{l} C\;=\;\sigma \left(W*\emptyset \left(P\left(I_{\left(x\text{\_}ray\right)}\right)\right)\;+\;b\right) \end{array}$$ (14)

The sigmoid function is written below,


$$\begin{array}{l} C=\frac{1}{1+\;e^{-\left(W*\emptyset \left(P\left(I_{\left(x\text{\_}ray\right)}\right)\right)\;+\;b\right)}} \end{array}$$ (15)

Where *C* is used to define the class label normal/fibrosis, σ is the sigmoid function, *W* denotes the weight matrix, and *b* is the bias function. ∅(*P*(*I*_(*x*_*ray*)_)) represented by a ResNet feature extractor. The bias function compares the ground truth with the predicted output of the similarity index and then produces the segmentation output for the input images. Pooling computes the average value of each small region or window, thereby reducing the spatial dimensions of features. Based on this, each layer produces segmentation images of different sizes and pixel counts. Many layers of the network are converted into a single resultant image from the pooled feature map process achieved by flattening. Multiple images associated with a single resultant are concatenated into a single feature vector, which is then passed through an activation function and ultimately classified using sigmoid activation, resulting in improved model performance and efficient segmentation of outcome images.

Figure 4 explains the proposed system with a changed network and layer. The dropout value was set to 0.5 to improve the performance of metrics, and this is used to help prevent overfitting by forcing the methods to learn multiple representations of layers, which is used in architecture. In ES-D-HED, we have added extra layers for detecting edges clearly to get perfect segmentation results to identify the performance of the disease. During the training period, half of the dataset will be active, and the remaining will be stable. Once the process is completed, the same process will repeat to check the remaining dataset to activate for better performance. This process happened for testing and validation in the same way. Finally, ES-D-HED provides distinct layers with different stages in the segmentation results for the corresponding input and ground truth images before flattening; it then achieves an accuracy of 9.85% for efficient segmentation and classification of PF.

Mahapackialakshmi and Jaffino (2024) described that an X-ray image can be harnessed for accurate segmentation and classification of lung diseases, including normal and PF detection, by integrating cutting-edge feature extraction methods with deep learning models. This future scope offers exciting opportunities for enhancing diagnostic precision and patient outcomes. This study has demonstrated superior classification performance through simulation-based experiments.

**Algorithm 1:** Lung segmentation and classification.

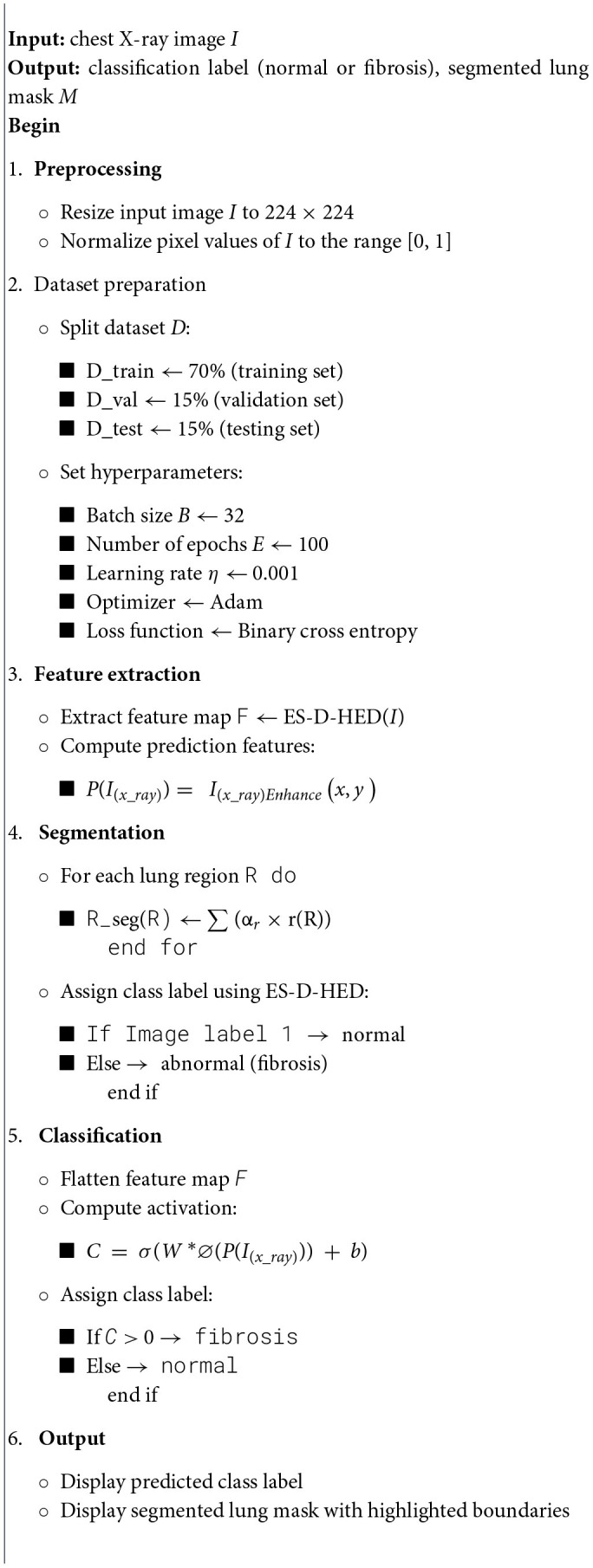

### 3.6 Performance evaluation metrics

Performance matrices are calculated by following the equation,

Accuracy: Acc is used to calculate the proportion of pixels or instances that are perfectly classified to the overall quantity of pixels and instances, which is given in Equation 15,


$$\begin{array}{l} Acc=\frac{TP\left(++\right)+TN\left(\pm \right)}{TP\left(++\right)+TN\left(\pm \right)+FP\left(\mp \right)+FN\left(--\right)} \end{array}$$ (16)

Where Acc means accuracy, TP(++) represents the true positive, TN(±) denotes the true negative, FP((∓)) represents the false positive, and FN(−−) denotes the false negative. The model accuracy measures the accuracy of edge map detection within the lung region and defines the segmented lung region that yields correct results.

Sensitivity: sensitivity (SENS) refers to correctly identifying patients with lung disease with a test or algorithm based on the ability to function. It measures the ratio of true positives (identification of lung disease) among all patients (with and without lung disease). The Equation 16 written below,


$$\begin{array}{l} SENS=\frac{TP\left(++\right)}{TP\left(++\right)+FN\left(--\right)} \end{array}$$ (17)

Specificity: specificity (SPEC) refers to correctly identifying patients without lung disease with a test or algorithm based on the ability to function. It measures the ratio of true positives (identification of lung disease) among all patients (with and without lung disease). The Equation 17 written below,


$$\begin{array}{l} SPEC=\frac{TN\left(\pm \right)}{TN\left(++\right)+FP\left(\mp \right)} \end{array}$$ (18)

Precision: precision (PREC) refers to correctly identifying patients with lung disease with a test or algorithm based on the ability of the test or algorithm to detect those who are positive. It measures the ratio of true positives (patients with lung disease) among all the patients with lung disease (only lung disease patients). The Equation 18 written below,


$$\begin{array}{l} PREC=\frac{TP\left(++\right)}{TP\left(++\right)+FP\left(\mp \right)} \end{array}$$ (19)

F1 score: F1 refers to the test- or algorithm-balanced measurement of both sens and prec. It provides a single value, i.e., 0 and 1. 1 denotes perfect accuracy, and 0 denotes perfect inaccuracy. The Equation 19 written below,


$$\begin{array}{l} F1=\frac{2^{*}\left(PREC^{*}SENS\right)}{\left(PREC+SENS\right)} \end{array}$$ (20)

For segmentation evaluation, quantitative measures were calculated, including dice similarity coefficient (DSC), intersection over union (IoU), and boundary *F*-score, using ground-truth masks annotated by the relevant clinical experts. The single dice coefficient is used to indicate spatial coincidence between the area of predicted and radiologist-defined fibrosis, and the boundary *F*-score is used to extract contour coincidence. This performance measure provides a better clinical interpretation to identify diseases.

Dice similarity coefficient: the dice similarity coefficient (DSC) is used to measure the intersection between two sets. It identifies the similarity between lung tissue segmentation and label segmentation. DSC Equation 20 is given below,


$$\begin{array}{l} DSC=\frac{2^{*}TP\left(++\right)}{\left(2^{*}TP\left(++\right)+FP\left(\mp \right)+FN\left(--\right)\right)} \end{array}$$ (21)

Intersection over Union (IoU): the Equation 21 represents the IOU.


$$\begin{array}{l} IoU=\frac{TP\left(++\right)}{\left(2^{*}TP\left(++\right)+FP\left(\mp \right)+FN\left(--\right)\right)} \end{array}$$ (22)

Boundary *F*-score (BF-score): the Equation 22 represents the BF score.


$$\begin{array}{l} BF=\frac{2^{*}PREC_{b}^{*}Recall_{b}}{PREC_{b}+Recall_{b}} \end{array}$$ (23)

Where *PREC*_*b*_ is denoted as boundary precision; *Recall*_*b*_ is denoted as boundary recall. Mask area ratio (MAR) is represented by Equation 23,


$$\begin{array}{l} MAR=\frac{\sum_{k=1}^{N}M\left(k\right)}{N} \end{array}$$ (24)

Entropy was calculated to quantify the texture complexity of the segmented lung region and is given below in Equation 24,


$$\begin{array}{l} Entopy=-\sum p\;log_{2}\;p \end{array}$$ (25)

From this, all parameters are calculated using mathematical expressions. This expression is calculated from input images of chest X-ray images. Parameters are measured individually, resulting in improved performance across all metrics. Each type (balanced and unbalanced) of image produced a different value of output. Each batch size (32) of the output was improved gradually to produce improved performance of the methods. These parameters are used to identify lung segmentation with and without disease. The accuracy of the identification statement can be evaluated by calculating the number of images affected by diseases and comparing it to the correct identifications.

## 4 Results and discussion

This section involves simulating the proposed deep learning model by experimenting with various hyperparameter combinations, including batch size, loss function, optimizer, number of epochs, and optimizer type, to optimize its performance. This section includes an ablation study to determine how various model components and hyperparameters affect performance, as well as results from evaluating the trained, fine-tuned ResNet152V2 model for the binary classification task. Batch size, learning rate, and layer modifications are just a few of the variables that may be changed to create a more reliable architecture with improved classification accuracy. An ablation study was carried out on these components to investigate this. *K*-Fold cross-validation was used to guarantee the model's robustness and dependability. CNN model was trained and validated to classify chest X-ray images as PF or normal. In this study, publicly available chest X-ray images of normal lungs, along with corresponding ground truth masks from the Kaggle dataset, were used. Healthy structural references were provided for these masks. The ES-D-HED framework was used to segment lung regions of all test images, including those of potentially diseased cases. Implemented a proxy evaluation by calculating a similarity score between the reference normal lung mask and the ES-D-HED result, because the ground truth segmentation mask for the diseased image was not available. A lower similarity score was interpreted as a possible suggestion of abnormalities. This study conducted a visual evaluation by showcasing the Grad-CAM heatmaps and ES-D-HED segmented edges produced by the classification model to support this conclusion.

The alignment between the detected edges and activation regions in Grad-CAM further validates the effectiveness of our unsupervised segmentation in identifying affected areas. The results of these assessments are described in detail in the ensuing subsections. System and software requirements are mentioned. All experiments were conducted on a Windows 11 (64-bit) system equipped with an Intel Core i7-13650HX processor, an NVIDIA GeForce RTX 3050 GPU, and 6 GB of memory. The proposed framework was implemented using Python and TensorFlow libraries. The batch size, optimizer type, learning rate, dropout rate, and number of units in the dense layer were among the hyperparameters considered for optimization. To reduce the negative validation accuracy, which served as the optimization objective function of the proposed model. Table 4 summarizes the hyperparameter configuration used to train and fine-tune the proposed ResNet152V2 Classification technique.

**Table 4:** Hyperparameter configuration used for the proposed training model.

Hyper-parameters	values
Batch size	32
Optimizer	Adam
Learning rate	0.001
Loss function	Binary_cross entropy
Epochs	100

### 4.1 Dataset sample visualization

**Figure 5:**
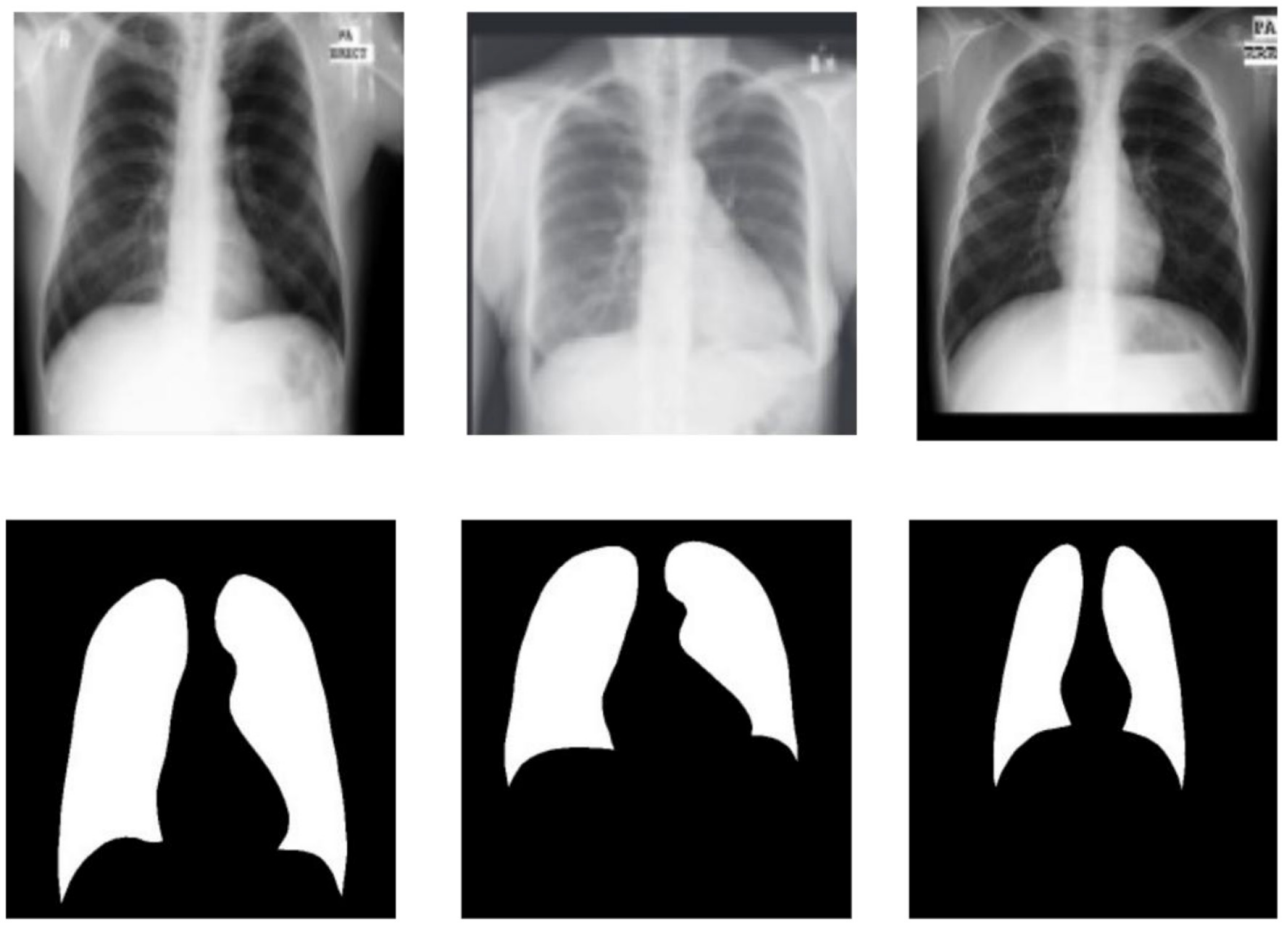
Sample images and corresponding labels.

Figure 5 shows the input and ground-truth images from the sample dataset (X-ray images). The dataset images are first trained using the ES-D-HED architecture, and then the preprocessed outputs are generated for all images. Subsequently, the processed input images are segmented to extract the lung region, and images with no lung diseases are identified. These sample images show various lung conditions from the dataset. Datasets have different sizes and image conditions.

### 4.2 Preprocessed image samples

**Figure 6:**
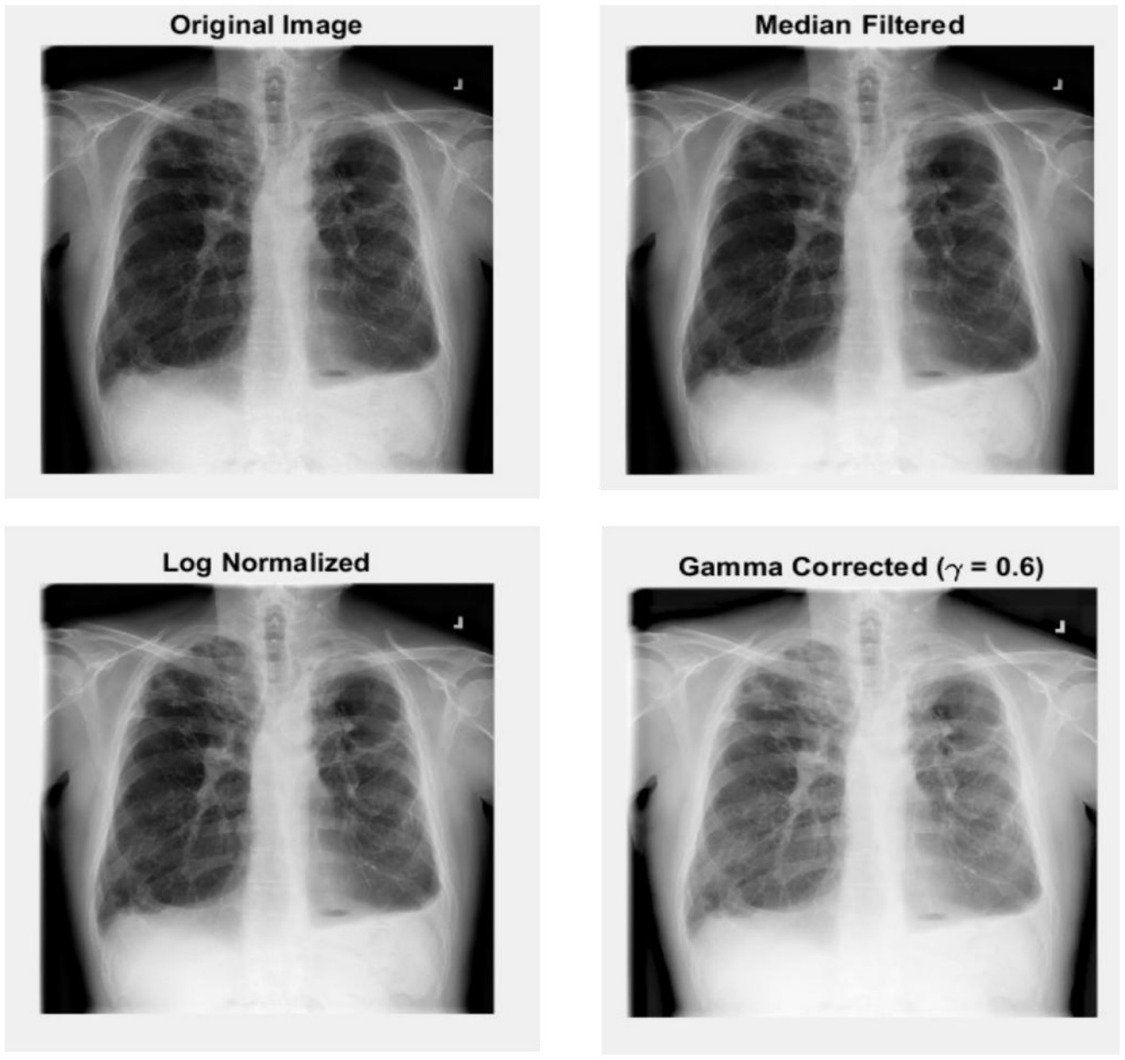
Representative preprocessing stages applied to chest X-ray images.

Figure 6 shows the preprocessing steps applied to input chest X-ray images before segmentation and classification. Median filtering was used to suppress noise artifacts while maintaining structural boundaries. Log normalization enhanced the reliability of the intensity distribution, while gamma correction enhanced the pulmonary regions with fibrosis and increased the contrast. The enhanced images were subsequently used as input for the proposed ES-D-HED segmentation and ResNet152V2 classification framework.

### 4.3 Segmentation performance

Segmentation of the lung is a very difficult task due to surrounding structures, including bones, and the brightness problem of the lung area. The CNN architecture effectively identifies the lung region by utilizing a combination of convolution, filter, pooling, and dropout layers, which collectively enhance the accuracy and efficiency of lung region detection. ES-D-HED approaches are used to segment the lung image for training and testing. Through training and testing on image data, a deep learning model can detect the edges of the lung region and the affected lung abnormalities. Segmentation of the lungs could easily identify normal and affected lung regions. The segmentation of fibrosis regions was quantitatively assessed against clinical expert-annotated masks. The value of dice coefficients indicates the spatial alignment with radiologist-defined structural areas.

**Figure 7:**
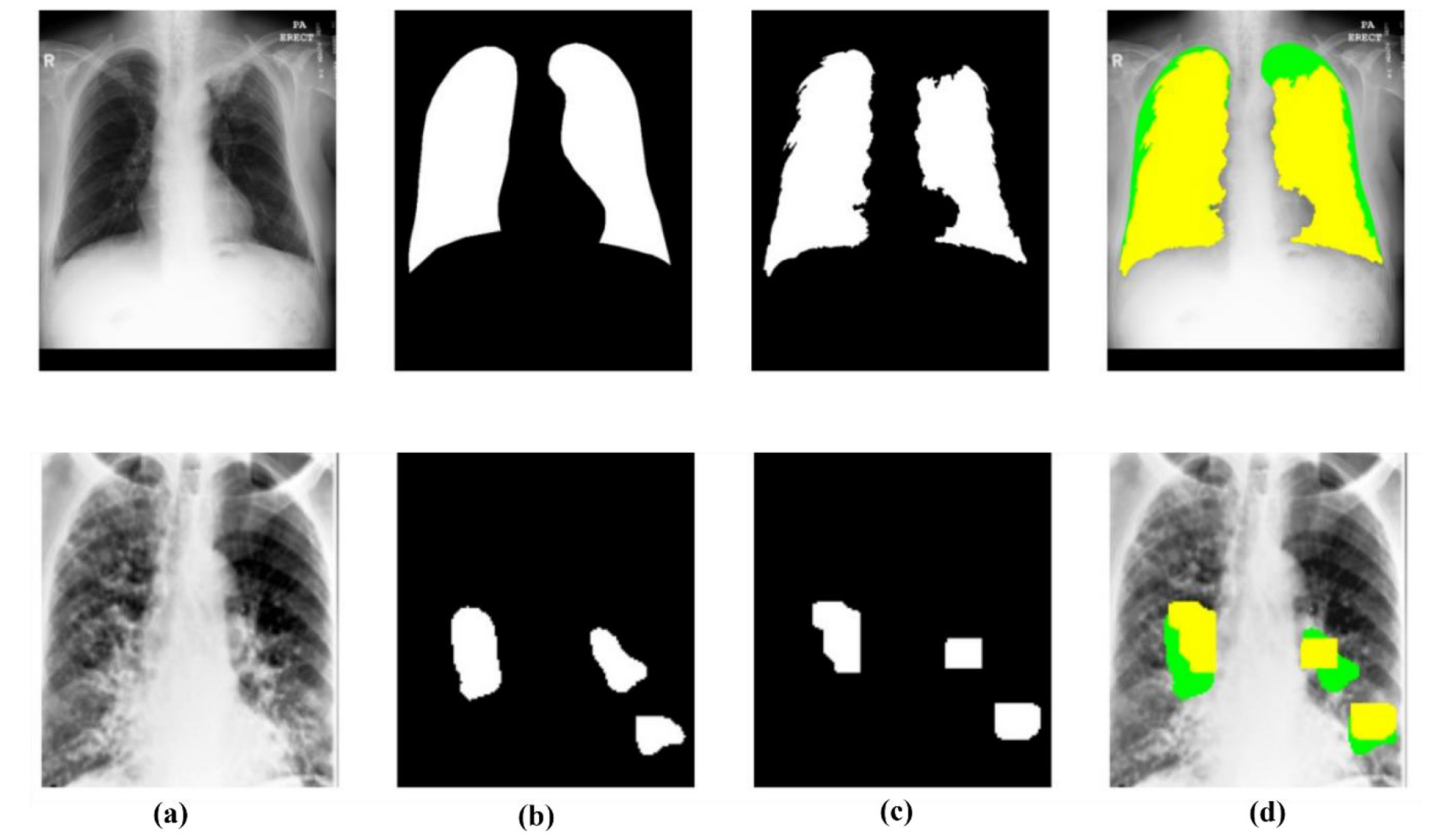
Segmentation results: normal and pulmonary fibrosis chest X-ray images showing the original image **(a)**, ground truth mask **(b)**, predicted mask **(c)**, and overlay comparison between ground truth (green) and predicted regions (yellow) **(d)**.

Figure 7 shows the qualitative segmentation outcomes obtained by the proposed method for both normal chest X-ray images and PF images. The primary column, Figure 7a, presents the original chest X-ray image; the second column, Figure 7b, denotes the ground truth mask; the third column, Figure 7c, displays the predicted segmentation mask; and the fourth column, Figure 7d, provides the overlay comparison between the ground truth and predicted results.

In the case of normal chest X-ray images, the ground truth mask corresponds to the annotated lung regions. The proposed method segments the lung fields by identifying the boundaries of the left and right lungs while also excluding nearby anatomical structures such as ribs and diaphragm. The predicted mask closely aligns with the ground truth, demonstrating that the model reliably captures the lung regions in normal images.

The ground truth masks for PF consist of lesion areas annotated by clinical experts and not the entire lung area. Lesion detection is not straightforward in the presence of heterogeneous lung textures and fibrotic opacities. Nevertheless, the proposed technique is successful in defining the areas of the most significant fibrosis throughout the lung fields. The overlay plot shows that there is a huge coincidence between the estimated areas of the lesions and the clinical expert annotations.

In the overlay visualization images, the yellow color regions denote the predicted segmentation outcomes, while the green color regions represent the ground truth mask. The observed overlap between these regions shows the usefulness of the proposed approach in identifying both lung regions in normal images and fibrosis lesions in PF images. The quantitative evaluation shows that the proposed method achieved a DSC of 0.904 for normal lung segmentation and 0.8434 for PF lesion segmentation.

Overall, the results confirm that the proposed approach can reliably perform lung region segmentation on normal chest X-rays and fibrosis lesion detection for PF images using the proposed model.

Table 5 summarizes the segmentation results of the proposed ES-D-HED framework for both normal and PF. In regular lung images, the accuracy of the segmentation procedure is tested qualitatively using reference ground-truth masks with improved dice and BF-scores, indicating a mapping between the region boundary of the images. In the case of PF, the performance of segmentation was measured on clinical expert-measured fibrosis masks of a representative subset. The values of Dice coefficients and BF-scores are used to assess spatial correspondence between the predicted segmentation maps to clinical radiologist defined fibrosis regions. Besides quantitative validation, there are structural measures in the form of edge density, mask area ratio, entropy, and boundary complexity reported to describe textural and morphological changes related to fibrotic progression. These structural measures are designed to be additional exploratory measures as opposed to the primary validation measures. Overall, the findings suggest strong performance in lung segmentation and substantive structural agreement in the presence of PF cases, while cautioning against interpreting the exploratory texture-based measures.

**Table 5:** Segmentation performance of the proposed ES-D-HED model.

Metrics	Normal (mean ±SD)	PF (mean ±SD)
Dice score	0.9040 ± 0.0131	0.8434 ± 0.0847
BF-score score	0.8817 ± 0.0977	0.7160 ± 0.3031
IoU	0.8248 ± 0.0230	0.7382 ± 0.1233
Edge density	0.0583 ± 0.0293	0.0127 ± 0.0091
Mask area ratio	0.2758 ± 0.0640	0.0579 ± 0.0293
Entropy	0.8345 ± 0.0947	0.3074 ± 0.1190
Boundary complexity	1.1213 ± 1.0434	2.5012 ± 0.6392

#### 4.3.1 Ablation study of ES-D-HED architecture

To quantitatively validate the architectural contributions of the proposed ES-D-HED model, an ablation study was conducted to evaluate four configurations: (i) baseline HED, (ii) HED with multi-scale fusion, (iii) ES-D-HED without fusion (dilated intermediate-output only), and (iv) the full ES-D-HED architecture integrating both fusion and dilation. The analysis was carried out using normal and PF images annotated by a clinical expert. Table 6 gives the ablation study of the proposed model.

**Table 6:** Ablation study of ES-D-HED architecture.

Model variant	Normal lung		Fibrosis region		Dice (PF lung)
	Dice	BF-score	Dice	BF-score	
Original HED	0.801	0.783	0.734	0.662	0.767
HED + fusion	0.824	0.794	0.772	0.697	0.789
ES-D-HED without fusion	0.819	0.788	0.789	0.702	0.791
ES-D-HED (proposed)	0.904	0.881	0.843	0.716	0.852

The ablation table demonstrates that the inclusion of multi-scale fusion (HED + fusion) enhances the performance of HED segmentation in comparison to the base HED across all metrics. In particular, the fibrosis region Dice score value becomes higher (0.734 to 0.772), and it gives better structural aggregation for the fusion of features. The proposed ES-D-HED architecture obtained the better dice score of the fibrosis region which increases 0.772 to 0.843 and the boundary *F*-score also increases from 0.697 to 0.716, shows the efficiency of the dilated intermediate-output branch to capture the contextual structural information, and to improve the continuity of the boundary in fibrosis lung regions. The initial observation of dilation, but not fusion, gives a dice score value of 0.789, which means that expanding the receptive field by itself enhances the contextual model of fibrosis structures. Nonetheless, the better performance of the ES-D-HED model gives the maximum dice score value of 0.843, which demonstrates that the fusion of multi-scale and dilated intermediate-output is complementary and which gives the better boundary continuity and structural representation.

Overall, the progressive performance improves across the model variants, quantitatively supports the architectural motivation of ES-D-HED, and confirms the contributions of both multi-scale fusion and dilated convolution to improve the segmentation performance.

### 4.4 Classification performance

Based on the graph, the training accuracy vs. loss value and validation accuracy vs. loss. It shows the improved performance of the proposed model. To estimate the performance of the proposed models, *K*-Fold (*K* = 5) cross-validation is employed. Initially, the dataset was collected from publicly available sources, and after preprocessing, the input data were labeled as abnormal or normal and split into 70% for training, 15% for validation, and 15% for testing. Using one fold as the test set and the remaining fold as the training set for iteration. The dataset is partitioned into 32 batches. Utilizing a large batch size can significantly reduce training time while maintaining enhanced performance

Based on the trained deep learning method to classify the feature-extracted region of lungs as normal and abnormal. After this process, the accuracy and loss functions are calculated. Accuracy increased as the same loss decreased, indicating improved performance of this model compared to the existing system. The proposed model is evaluated and achieves high accuracy on the given dataset. Initially, the training model achieved an accuracy of 0.716 with a loss of 1.949, and the validation achieved an accuracy of 0.5 with a loss of 0.225. In the middle stage, it achieves only 0.939 accuracy on the training set with a loss of 0.199, while validation accuracy is 0.73 and the loss is 0.43. The highest accuracy of the training model reached the value 0.985 with a low loss of 0.15, and the validation performance reached 0.94 with a loss value of 0.273. This training and validation process provides enhanced results compared with the existing model.

**Figure 8:**
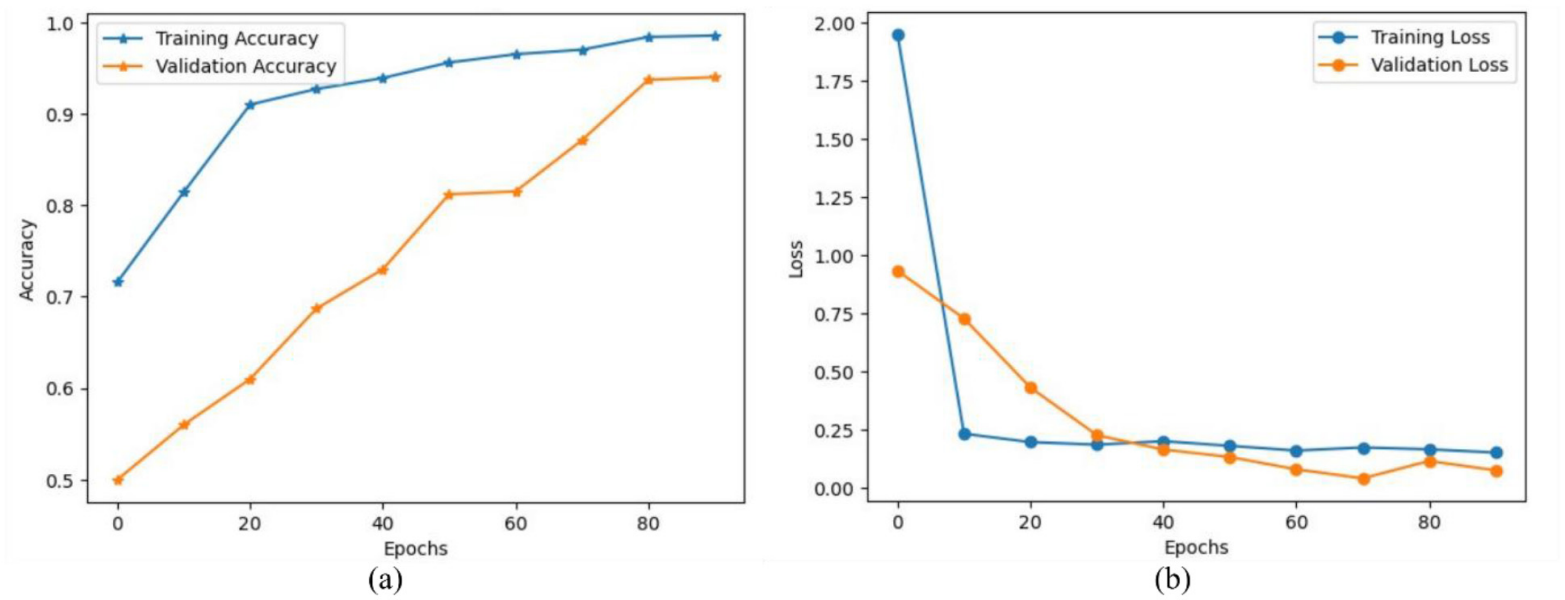
Training and validation performance curves of the proposed model classification framework during model optimization. **(a)** Accuracy and **(b)** loss.

Figure 8 shows the accuracy of training and validation, which depicts the model over a sequence of epochs. Among 100 epochs, selected 10 enhanced values and plotted the graph. Training accuracy gives enhanced performance. The validation value fluctuates due to data quality issues stemming from imbalanced classes, leading to inconsistent performance and highlighting the need for effective class balancing strategies to improve model reliability. Figure 8a shows that the blue line indicates model performance improves as the model learns from the dataset images. The next orange line indicates that the data is unbalanced for measuring the model generalization; it is crucial to monitor the parameters to avoid overfitting. Here, training accuracy increases while the validation accuracy is stable. In this validation, accuracy is used to identify the disease in lung X-ray images. Another Figure 8b shows the loss function of training and validation levels that could be decreased potentially, and the performance of the loss function is improved, and cross-fold validation was employed to enhance the generalization capability of the proposed model.

Table 7 represents the comparative analysis of classification performance. Different deep learning models were used to classify images as normal or abnormal. Among these various models, the ResNet-152V2 gives the improved performance.

**Table 7:** Performance comparison of classification on test images.

Model	Accuracy	Sensitivity	Specificity	Precision	F1 score
DenseNet-121	0.96	0.956	0.964	0.964	0.959
DenseNet-201	0.964	0.975	0.953	0.952	0.9633
VGG 16	0.974	0.96	0.968	0.968	0.9639
ResNet-50	0.978	0.98	0.976	0.976	0.977
**Proposed model ResNet-152V2**	**0.986**	0.98	**0.992**	**0.992**	**0.985**

The improved values are highlighted in bold.

**Figure 9:**
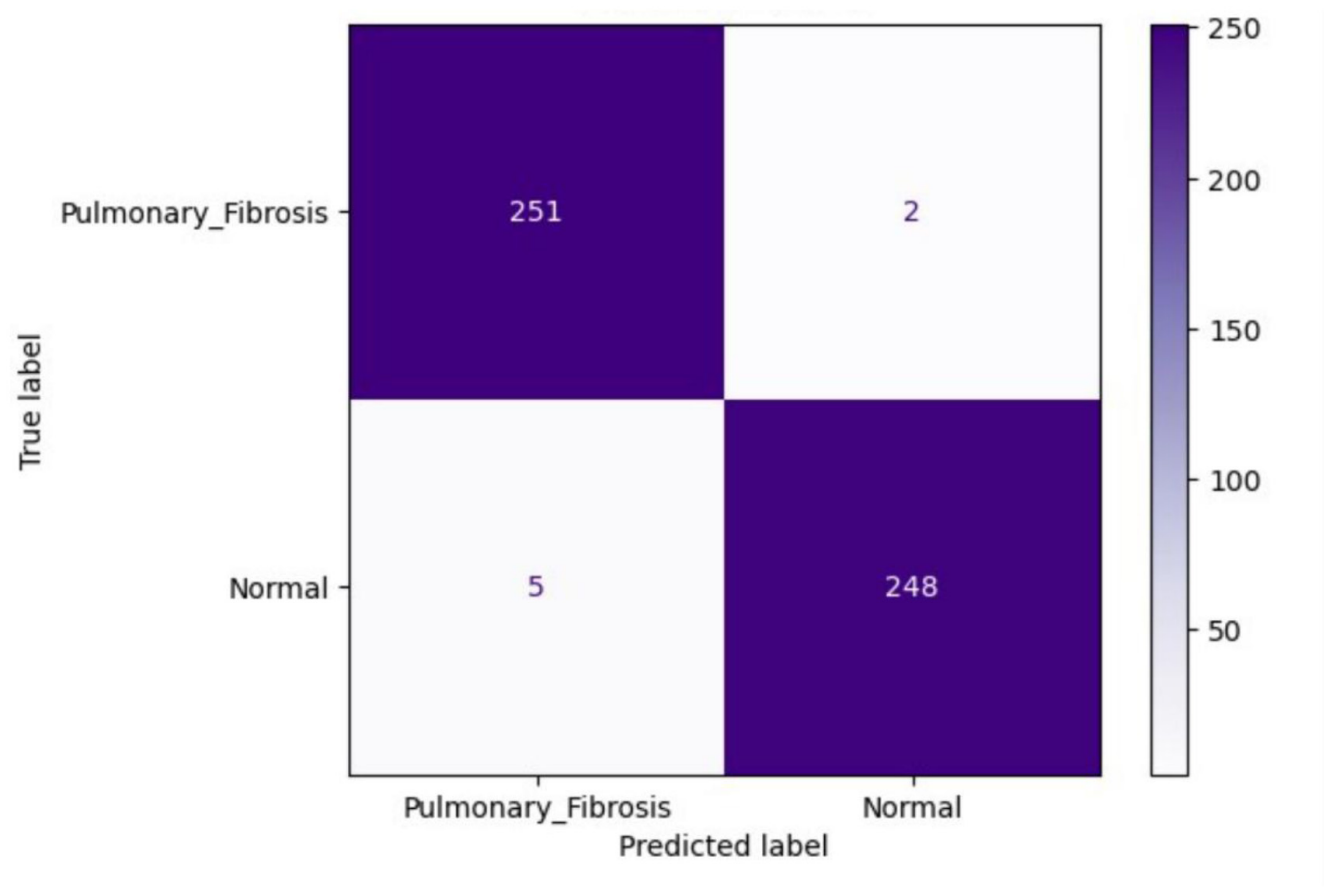
Confusion matrix of proposed model.

**Figure 10:**
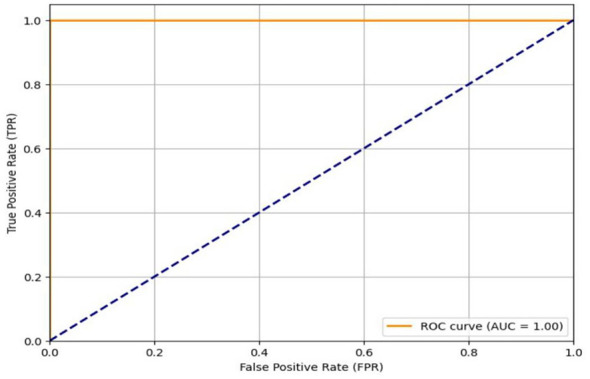
ROC curve of the proposed model.

*Confusion matrix*: the evaluation of the trained ResNet152V2 model was directed on classification accuracy, sensitivity, Specificity, precision, and F1-score using 506 X-ray images from the test dataset. The performance of the proposed model was evaluated by identifying perfectly and imperfectly classified data using a confusion matrix (CM). As shown in Figure 9, the model could recognize 248 normal chest X-rays, missing five in the normal class as fibrosis. The model correctly recognized 251 abnormal images in the PF class, and two X-ray images were incorrectly recognized as normal. The effectiveness of the proposed PF detection model is demonstrated by its ROC curve in Figure 10, which illustrates the model's classification performance.

**Figure 11:**
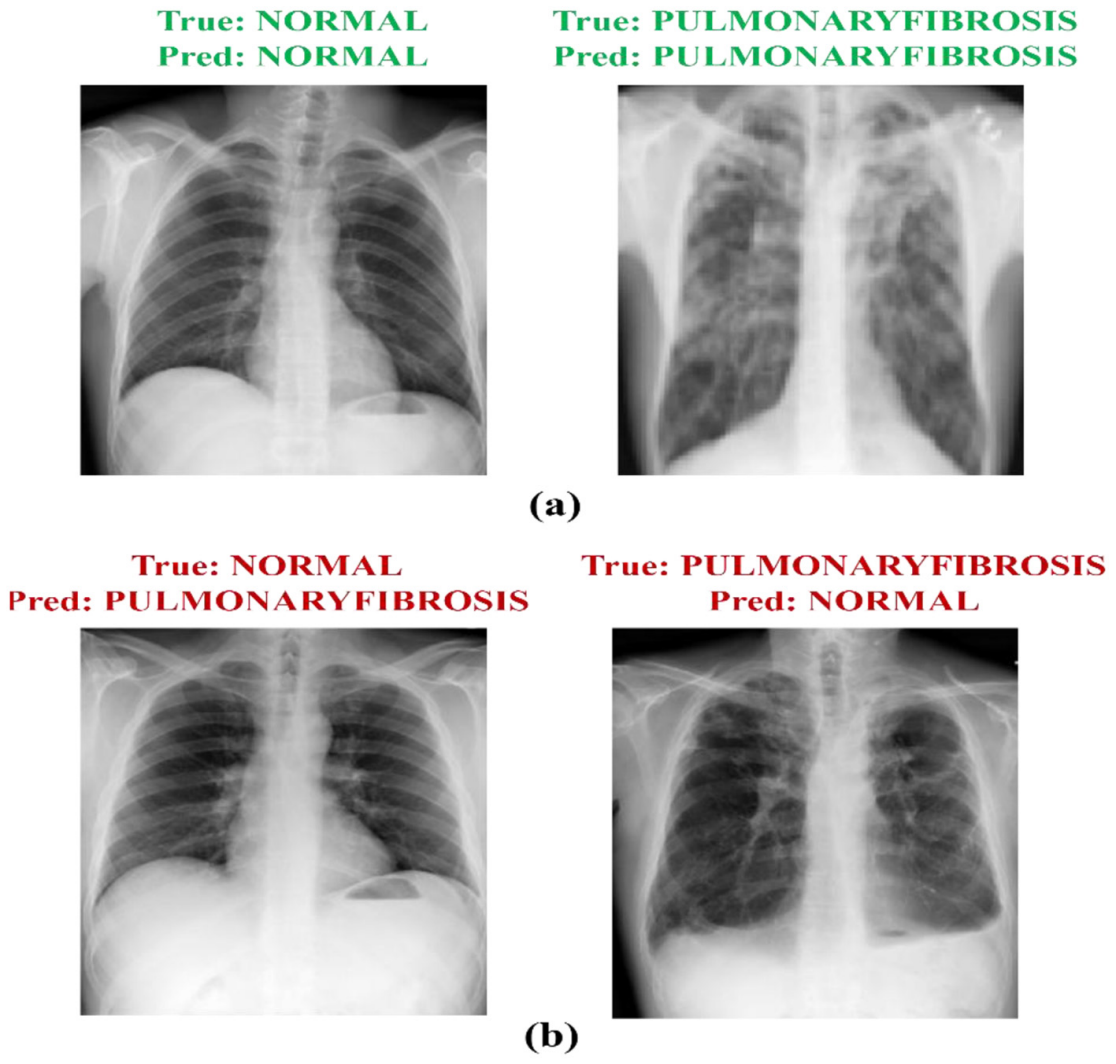
Classification result of the proposed model. **(a)** Correctly classified results and **(b)** misclassified results.

To present the classification results shown in Figure 11, representative samples of chest X-ray images were chosen from the validation dataset after preprocessing and the ES-D-HED-based segmentation process. The segmented lung regions were then fed into the fine-tuned ResNet152V2 classification model, which generated the normal or PF class labels using the learned feature representations. Figure 11a shows the correctly classified results, while Figure 11b illustrates the misclassified results.

The developed framework demonstrated competitive performance with respect to recent models of X-ray-based PF detection. Specifically, the obtained accuracy of 98.6% is higher than that of a number of previous methods based on MobileNetV2 and Inception-ResNet models. This can be explained by the fact that the ES-D-HED segmentation module enables better representation of the structural boundaries before classification. The development of fibrosis tends to create distorted, irregular shapes in the stitches. Furthermore, the enlarged intermediate branch results enhance the contextual edge modeling and enhance the ability of the ResNet152V2 classifier to discriminate the features. In contrast to the classification methods, the proposed model also uses boundary-sensitive structural data, as evidenced by Grad-CAM alignment with fibrosis regions annotated by clinical experts. These results indicate that edge-strengthened segmentation positively affects diagnostic performance. Exceptional performance on a singular dataset may indicate dataset-specific attributes or intrinsic class separability. Although patient level separation was implemented to prevent data leaking, potential for dataset bias or overfitting to distribution specific characteristics cannot be entirely ruled out. To overcome the overfitting issue, *k*-fold cross-validation is included in this research. The large-scale multi-center validation and prospective clinical trials are used to evaluate applicability in the real world and integration into clinical decision support systems.

*K-fold cross validation*: to prevent the overfitting and thoroughly evaluate proposed model performance, employed five-fold cross-validation. By distributing the dataset into k subsets and iteratively training the model on *k* – 1 subsets while using the remaining subset for validation, this technique enables a robust evaluation. A thorough evaluation of the model's performance is then obtained by averaging the outcomes from each iteration. The dataset was separated into five subgroups for the five-fold cross-validation. Using four subsets for training and a different subset for validation, the model was trained and validated five times. The average values indicate that the model's performance improved across all folds, while the low standard deviations (±0.0121 for accuracy and ±0.0075 for loss) indicate that the model exhibited stable and consistent behavior during cross-validation. Table 8 summarizes the findings.

**Table 8:** Five-fold validation performance.

Fold	Accuracy	Loss
1	0.9683	0.1013
2	0.9744	0.0829
3	0.9885	0.0830
4	0.9978	0.0980
5	0.9993	0.0883
Mean	0.9860	0.0907
Std dev	±0.0121	±0.0075

**Figure 12:**
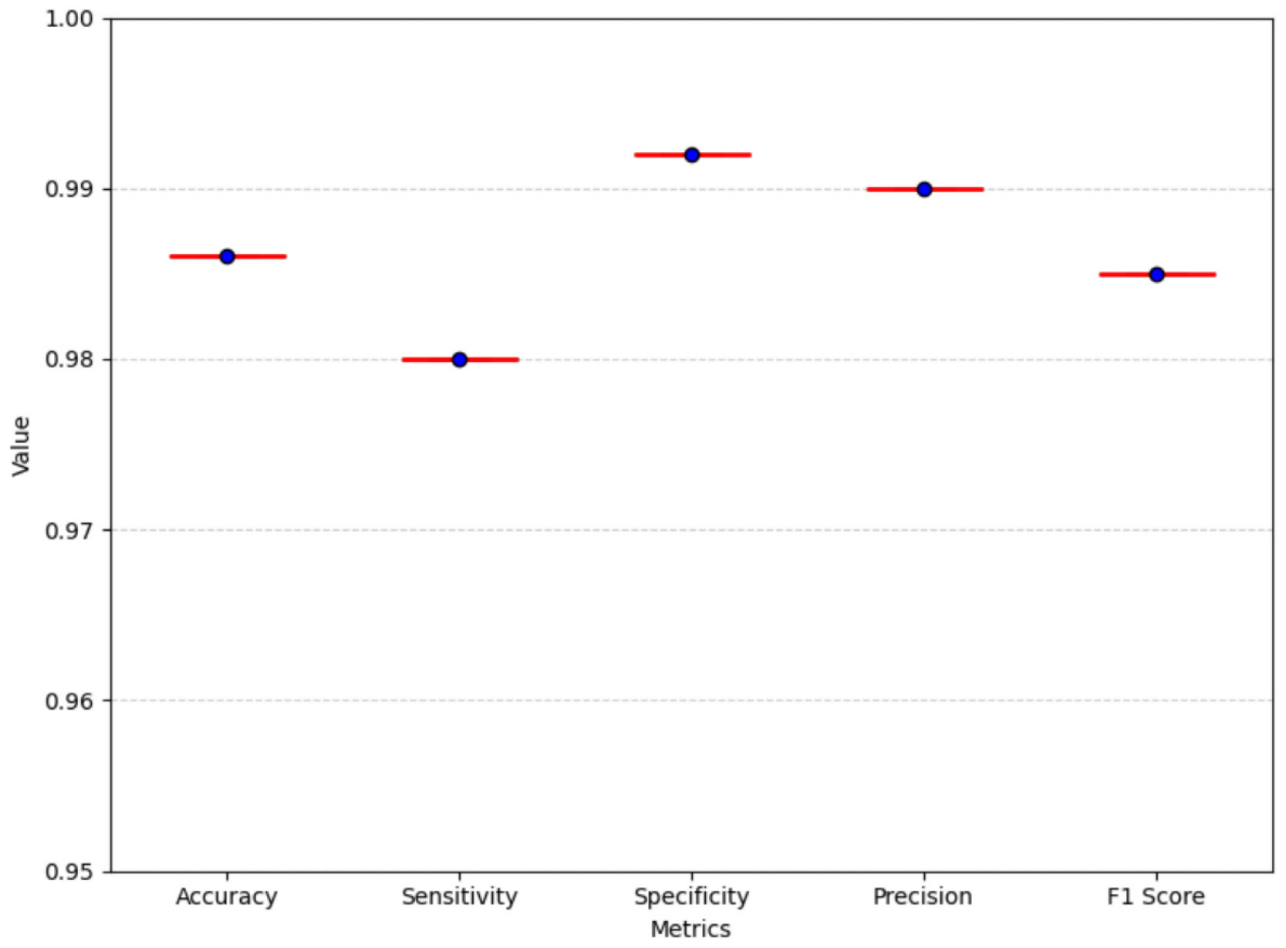
Performance metrics of the proposed classification method.

Table 9 shows the comparison and average value of all the metrics, the total number of X-ray images is the input collected to the data set. The dataset is used to train and test the proposed model. The proposed model has achieved an accuracy is 0.986, Sensitivity is 0.98, Specificity is 0.9929, Precision is 0.99, and F1 score is 0.985. The average performance metrics are shown in Figure 12.

**Table 9:** Average value of performance metrics.

Model	Accuracy	Sensitivity	Specificity	Precision	F1 score
Proposed model	0.986	0.98	0.992	0.99	0.985

**Figure 13:**
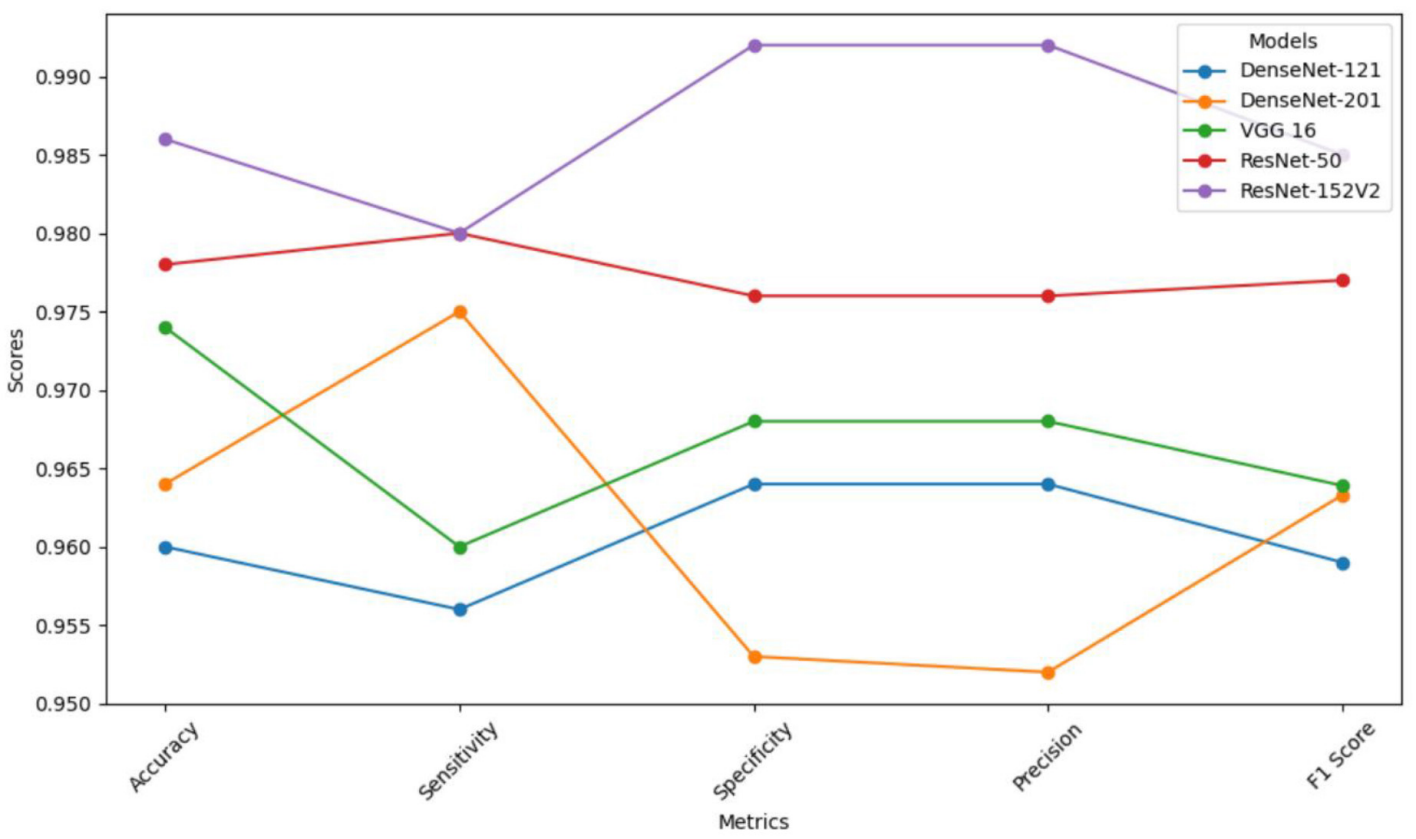
Performance of classification with all models.

Figure 13 shows the comparative performance of all classification models. Each parameter has a different color to indicate different values and also compared with all metrics, the specificity value (0.992) is high, and the sensitivity (0.98) value is low compared to other metrics too.

#### 4.4.1 Ablation study of classification

##### 4.4.1.1 Case I: layer changes

The input layer resizes the image dimensions to 224 × 224 × 1 and preprocesses the input image to enhance the quality of the region of the X-ray images. The pre-trained model ResNet50V2 base is stable, and the added classification layers are trained and given a minimum level of performance before fine-tuning conditions so for better performance some changes are done in the model. That's the last 15 unfrozen layers, and the learning rate was adjusted. After changes model gives an improved performance with 98.5% classification accuracy after fine-tuning conditions. Table 10 summarizes the findings.

**Table 10:** Performance of the added layer.

Configurations	Accuracy	Sensitivity	Specificity	Precision	F1 score	Findings
Baseline (ResNet152V2)	0.943	0.910	0.950	0.947	0.930	Accuracy dropped
Median filter + gamma (0.6) + dense (256) + dropout (0.4)	0.960	0.928	0.968	0.959	0.941	Accuracy dropped
Median filter + gamma (0.6) + dense (512) + dropout (0.5) + BatchNorm	0.986	0.980	0.992	0.992	0.985	Accuracy enhanced

##### 4.4.1.2 Case II: choosing the best batch size

Batch sizes of 16, 32, and 64 were used for training. Batch size 16 gave low-level performance, and batch size 64 gave variations in speed and memory performance. However, size 32 balanced speed, memory, and generalization, and then yielded the best performance for the proposed model compared to these three batch sizes. Table 11 summarizes the findings.

**Table 11:** Modification of batch size.

Configurations	Accuracy	Sensitivity	Specificity	Precision	F1 score	Findings
16	0.96	0.95	0.98	0.956	0.94	Accuracy dropped
32	0.986	0.98	0.992	0.99	0.985	High accuracy
64	0.986	0.98	0.992	0.992	0.985	Similar accuracy

##### 4.4.1.3 Case III: electing the best learning rate

Choosing the correct learning rate is challenging because, during training, the learning rate value may automatically change to optimize performance. To avoid issues like overshooting and to ensure the model achieves improved performance, careful tuning and monitoring of the learning rate are necessary. Here, learning rates of 0.0001 and 0.01 were tested. A learning rate of 0.001 provided better accuracy for the proposed model. Table 12 provides a summary of the findings.

**Table 12:** Modification of learning rate.

Configurations	Accuracy	Sensitivity	Specificity	Precision	F1 score	Findings
0.0001	0.95	0.94	0.96	0.93	0.92	Accuracy dropped
0.001	0.986	0.98	0.992	0.992	0.985	High accuracy
0.01	0.98	0.96	0.976	0.968	0.97	Accuracy dropped

### 4.5 Grad-CAM explainability visualization

**Figure 14:**
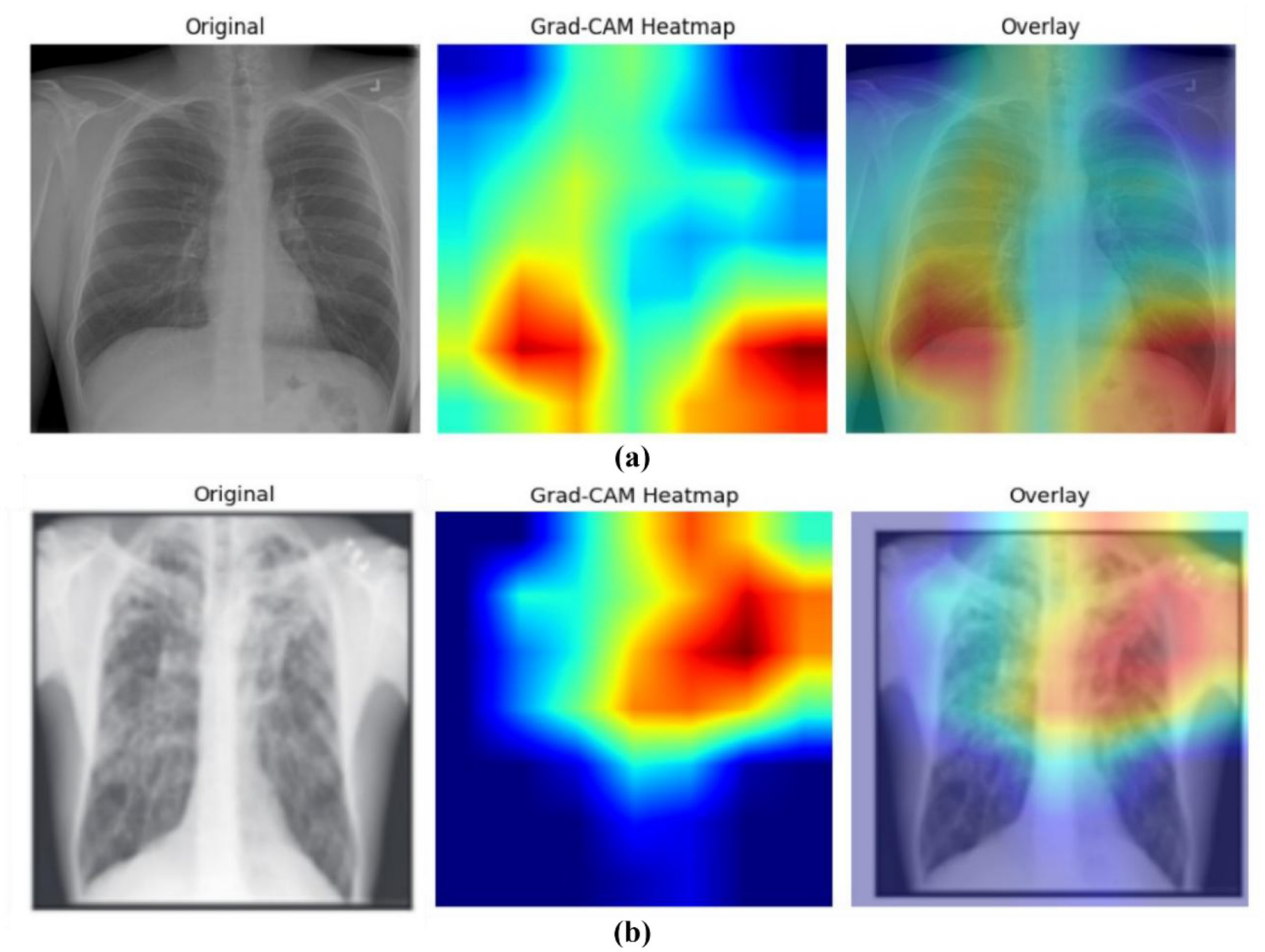
Grad-CAM visualization results of the proposed model classification framework showing activation patterns for normal **(a)** and pulmonary fibrosis **(b)** chest X-ray images.

Gradient-weighted class activation mapping (Grad-CAM) was used to understand the decision-making process of the classification model. Grad-CAM heatmaps indicate parts of the image that make the largest contribution to the classification result. In Figure 14, the red and yellow parts represent the increased activation, and the blue parts denote decreased contribution to the decision of the models. The Grad-CAM visualization of Figure 14a denotes that the normal chest X-ray images showed relatively weaker and more diffuse activation with no prominent localization in the fibrosis-sensitive lung regions. Conversely, Figure 14b denotes the cases of PF that showed clustered activation in structurally abnormal regions of the lung, suggesting that the proposed ResNet152V2 framework was able to learn discriminative features related to fibrosis and classify the cases.

### 4.6 Comparative analysis with existing methods

Table 13 compares the performance of the existing model with the proposed methodology. The table shows that the proposed model performs well compared to the current deep-learning CNN model used for lung condition segmentation, which classifies cases as normal or PF. It provides an analysis of the precision and publication year of various X-ray image analysis methods. Bharati et al. (2020) presented a VDSNet model that achieved an accuracy of 0.5858 with an X-ray image dataset. Souid et al. (2021) provided 0.96 accuracies with image data, Li et al. (2022) described that ResNet v2 achieved 0.93% of precision with chest x-ray images, Amador et al. (2024) presented a ResNet50-ViT model that achieved 0.96% of accuracy, Fu et al. (2025) described that MaxVit produced accuracies of 0.97, and the proposed model provided 0.986 accuracies. Among all the methods, the proposed model, ResNet152V2, achieves the highest accuracy in X-ray image analysis.

**Table 13:** Comparison of the proposed methodology with the existing methodology.

References	Data type	Model	Accuracy
Souid et al. (2021)	X-ray	MobileNet V2	0.9600
Li et al. (2022)	X-ray	Inception-ResNet v2 CNN architecture	0.9300
Fu et al. (2025)	X-ray	MaxVit	0.9700
Bharati et al. (2020)	X-ray	VDSNet	0.5858
Amador et al. (2024)	X-ray	ResNet50 and vision transformers (ViT; fusion model)	0.9600
Proposed method	X-ray	ResNet152V2 architecture with ES-D-HED	0.9860

**Figure 15:**
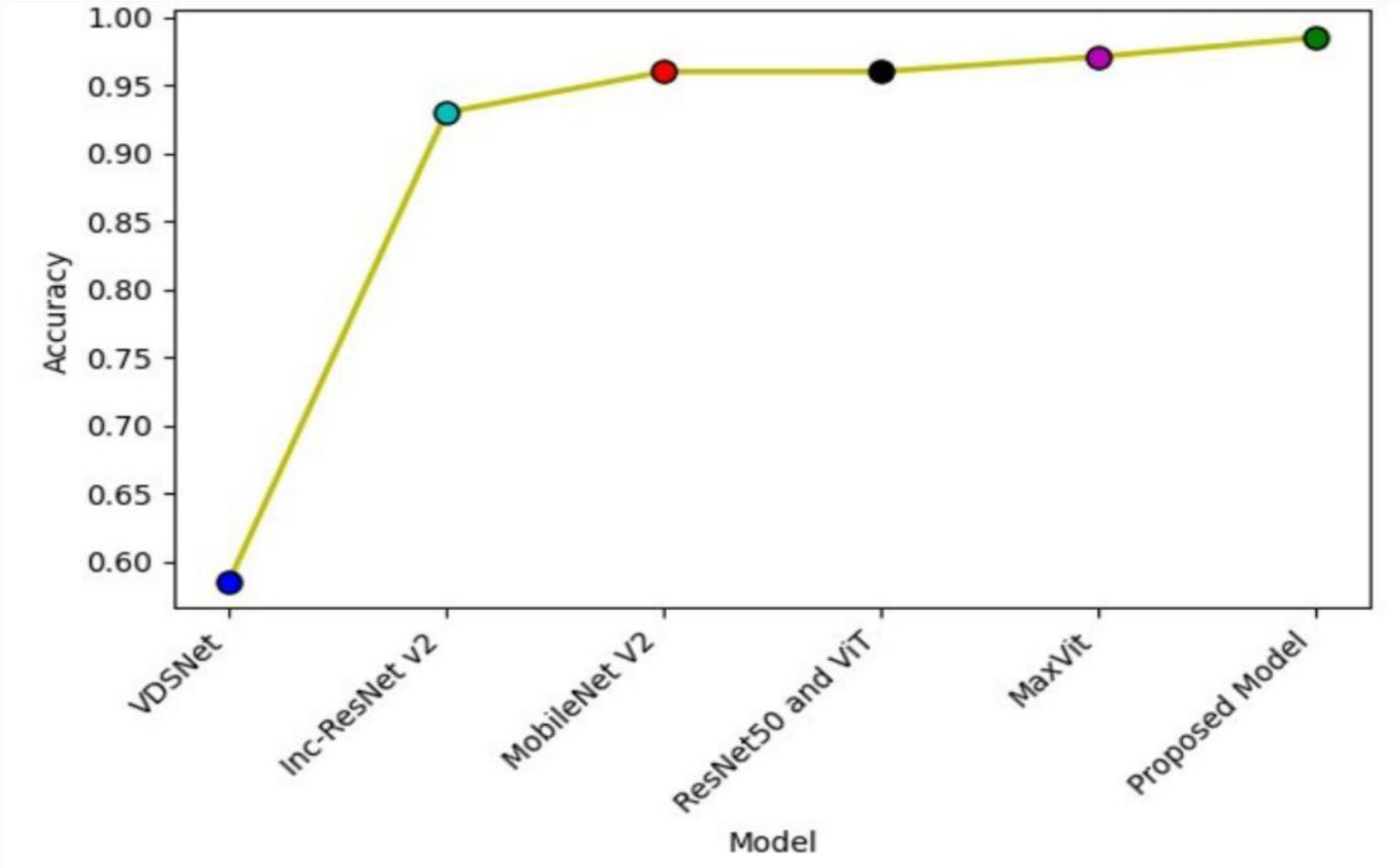
Comparison of the proposed methodology with the existing methodology.

Figure 15 compares all existing models with the proposed model. The comparison of model accuracies reveals a significant variance in performance among the different architectures for images of different sizes. This comparison provides a general performance context for the proposed method with PF disease detection and prediction, which is also a focus of existing studies. The VDSNet model achieved a relatively low accuracy of 0.5858, whereas the MobileNet V2 model showed a substantial improvement to 0.96. However, the Inception-ResNet v2 CNN Architecture surpassed both with an impressive accuracy of 0.930. The ResNet50-ViT model yielded an accuracy of 0.96, while the MaxViT model recorded 0.97. Notably, the proposed Model ResNet152V2 architecture emerged as the top performer, achieving an exceptional accuracy of 0.986. This stark contrast in performance underscores the importance of architecture selection and the potential benefits of incorporating advanced techniques, such as ES-D-HED, into CNN designs.

### 4.7 Limitations of the study

The research studies mainly focus on binary classification (normal vs. PF) and do not include any severity grading or distinctions among interstitial lung diseases. The analysis is used only for X-ray imaging.

## 5 Conclusion

This research presented an edge-strengthened deep learning framework combining ES-D-HED-based structural segmentation with ResNet152V2 classification to detect PF in chest X-ray images. The suggested segmentation module improves boundary-sensitive structural modeling, which was quantitatively tested on clinical experts' labeled fibrosis masks on a representative subset. The classification model achieves the best results on a curated subset of the NIH Chest X-ray dataset, with an accuracy of 98.6%. In addition to validating quantitative segmentation, other secondary measures describing morphological irregularities related to fibrosis were evaluated. A combination of segmentation and classification frameworks enables valuable structural alignment and explanatory decision support via Grad-CAM visualization. Although promising, the framework was tested only on a single dataset that needs to be externally multicentered to ensure generalizability. Additionally, chest X-ray imaging is intrinsically limited compared with HRCT in the ability to identify small fibrotic changes. Consequently, the suggested system is to be perceived as a screening support and methodological system rather than an independent diagnostic instrument. Future work will focus on extending the framework to multi-class interstitial lung disease classification, fibrosis severity grading, and integration with multi-modal imaging data to improve clinical applicability.

## Data Availability

The original contributions presented in the study are included in the article/supplementary material, further inquiries can be directed to the corresponding author.
